# Innovation in unruptured intracranial aneurysm coiling: At which price or efficacy are new technologies cost-effective?

**DOI:** 10.1371/journal.pone.0255870

**Published:** 2021-08-09

**Authors:** David Ben-Israel, Brooke L. Belanger, Amin Adibi, Muneer Eesa, Alim P. Mitha, Eldon Spackman

**Affiliations:** 1 Department of Clinical Neurosciences, University of Calgary, Calgary, Alberta, Canada; 2 O’Brien Institute for Public Health, University of Calgary, Calgary, Alberta, Canada; 3 Hotchkiss Brain Institute, University of Calgary, Calgary, Alberta, Canada; 4 Collaboration for Outcomes Research and Evaluation, Faculty of Pharmaceutical Sciences, University of British Columbia, Vancouver, Canada; 5 Department of Radiology, University of Calgary, Calgary, Alberta, Canada; 6 Department of Community Health Sciences, University of Calgary, Calgary, Alberta, Canada; Barrow Neurological Institute, UNITED STATES

## Abstract

**Background:**

Unruptured intracranial aneurysms (UIA) are increasingly being treated by endovascular coiling as opposed to open surgical clipping. Unfortunately, endovascular coiling imparts an approximate 25% recanalization rate, leading to additional procedures and increased rupture risk. While a new health technology innovation (HTI) that reduces this recanalization rate would benefit patients, few advancements have been made. We aim to determine whether cost-effectiveness has been a barrier to HTI.

**Methods:**

A probabilistic Markov model was constructed from the healthcare payer perspective to compare standard endovascular treatment of UIA to standard treatment plus the addition of a HTI adjunct. Costs were measured in 2018 USD and health outcomes were measured in quality-adjusted life-years (QALY). In the base case, the HTI was a theoretical mesenchymal stem cell therapy which reduced the aneurysm recanalization rate by 50% and cost $10,000 per procedure. All other model inputs were derived from the published scientific literature.

**Results:**

Based on the model results, we found that for a given HTI price (*y*) and relative risk reduction of aneurysm recanalization (*x*), the HTI was always cost-effective if the following equation was satisfied: *y* ≤ 20268 ∙ *x*, using a willingness-to-pay threshold of $50,000 per QALY. The uncertainty surrounding whether an aneurysm would recanalize was a significant driver within the model. When the uncertainty around the risk of aneurysm recanalization was eliminated, the 10-year projected additional benefit to the United States healthcare system was calculated to be $113,336,994.

**Conclusion:**

Cost-effectiveness does not appear to be a barrier to innovation in reducing the recanalization rate of UIA treated by endovascular coil embolization. Our model can now be utilized by academia and industry to accentuate economically feasible HTI and by healthcare payers to calculate their maximum willingness-to-pay for a new technology. Our results also indicate that predicting a patient’s baseline risk of aneurysm recanalization is a critical area of future research.

## Introduction

In the last two decades, few approaches in disease management have changed more dramatically than the treatment of intracranial aneurysms. Numerous landmark publications have clearly demonstrated the viability of endovascular coil embolization for both ruptured and unruptured intracranial aneurysms (UIA) [1–3]. As coil embolization has become standard practice, an increasing number of coiling procedures are occurring each year [4] and a higher proportion of UIA are being treated [5]. After an aneurysm is filled with coils, while it is considered completely obliterated, there is a risk that over time the coils will become more compact and allow blood to refill a portion of the aneurysm [6]. This phenomenon is referred to as aneurysm recanalization, which is of concern since recanalized aneurysms have the potential to rupture, causing significant morbidity and mortality.

Multiple studies have shown that the coiling of UIA is cost-effective compared to open surgical clipping and conservative management [7–10]. Despite its comparable cost-effectiveness to surgery, coil embolization imparts a high recanalization rate of approximately 25% over 6 years, leading to repeat procedures and increased risk of aneurysm rupture [6]. Interestingly, none of the previous cost-effectiveness analyses have taken aneurysm recanalization into account. Furthermore, little has been published on adjunctive strategies to reduce the recanalization rate seen with the use of conventional Gugliemi coils. This begs the question of whether economic barriers have prevented innovation in this field. There exists a dilemma in HTI wherein the lack of preliminary efficacy data prevents the exploration of cost-effectiveness. Without a sense of whether an innovation will be adopted by healthcare payers, industry may be hesitant to invest in research and development. A theoretical economic model which circumvents this dilemma by exploring all efficacy levels may help encourage industry to pursue HTI.

Mesenchymal stem cell therapy has been proposed as a health technology innovation (HTI) to reduce the rate of aneurysm recanalization and the need for retreatment [11]. While this study maintains a general perspective and can be applied to any HTI, we have incorporated the use of mesenchymal stem cells in our base case in order to demonstrate how this economic analysis can evaluate any nascent innovation. Mesenchymal stem cells are immune evasive and can undergo multi-lineage differentiation which allows for targeting inflammatory conditions without triggering a significant immune response [12]. Currently, a variety of medical applications are under active exploration including multiple sclerosis, osteoarthritis, and cardiovascular tissue repair [13–16]. Once introduced to mesenchymal stem cells, UIA and abdominal aortic aneurysms have both demonstrated improvement in healing [17, 18].

In this study, we develop the first economic model for coiled UIA that incorporates the occurrence of aneurysm recanalization. We use this model to explore if the addition of a HTI that reduces the recanalization rate of coiled UIA is cost-effective. Our aim was to determine whether cost-effectiveness is a barrier to innovation, and if appropriate, to have this study serve as an impetus for academia, industry, and government investment.

## Methods

To perform this cost-effectiveness analysis, we constructed a Markov model, from a healthcare payer perspective, that simulates the flow of patients through the healthcare system after being treated with coil embolization for an UIA. A Markov model was chosen because it is the ideal model type to follow patients with chronic diseases who may transition between multiple health states over time. All costs are presented in 2018 United States dollars (USD) and were converted using the CCEMG–EPPI-Centre Cost Converter (Version 1.6) when necessary [19]. Costs and quality-adjusted life-years (QALY) were both discounted at 1.5% as per national guidelines [20, 21].

We performed this analysis in accordance with the Consolidated Health Economic Evaluation Reporting Standards statement [22] and Second Panel on Cost-Effectiveness in Health and Medicine [21]. Both checklists are available in S1.01 and S1.02 Tables in S1 File. We stress that this analysis is theoretical, based on results drawn from the broad scientific literature. To gain a better sense of the economic impact a HTI would have on a specific healthcare setting, regional- and HTI-specific parameters would need to be used. Therefore, in keeping with the Transparency and Openness Promotion guidelines, we offer to repeat our analysis for new HTI, specific healthcare systems, or individual institutions [23]. Requests that provide parameter values and distributions may be submitted to the corresponding author.

### Markov model structure

In Markov models, patients exist in one of several mutually exclusive *states*. Patients may *transition* from one state to another during each *cycle* of the model. In our model, six-month cycle lengths were chosen to reflect the longest expected amount of time between the diagnosis of aneurysm recanalization and management. The model was run for 60 cycles (30 years), which was felt to be more appropriate than running the model for the remainder of a patient’s lifespan. Firstly, it was felt that beyond 30 years, any benefit from the HTI would have been realized. Additionally, as patients age, especially beyond 75, their other medical conditions begin to play a larger role in their overall functional status and life expectancy. Moreover, older patients may no longer be candidates or wish to pursue endovascular therapy.

**Fig 1:**
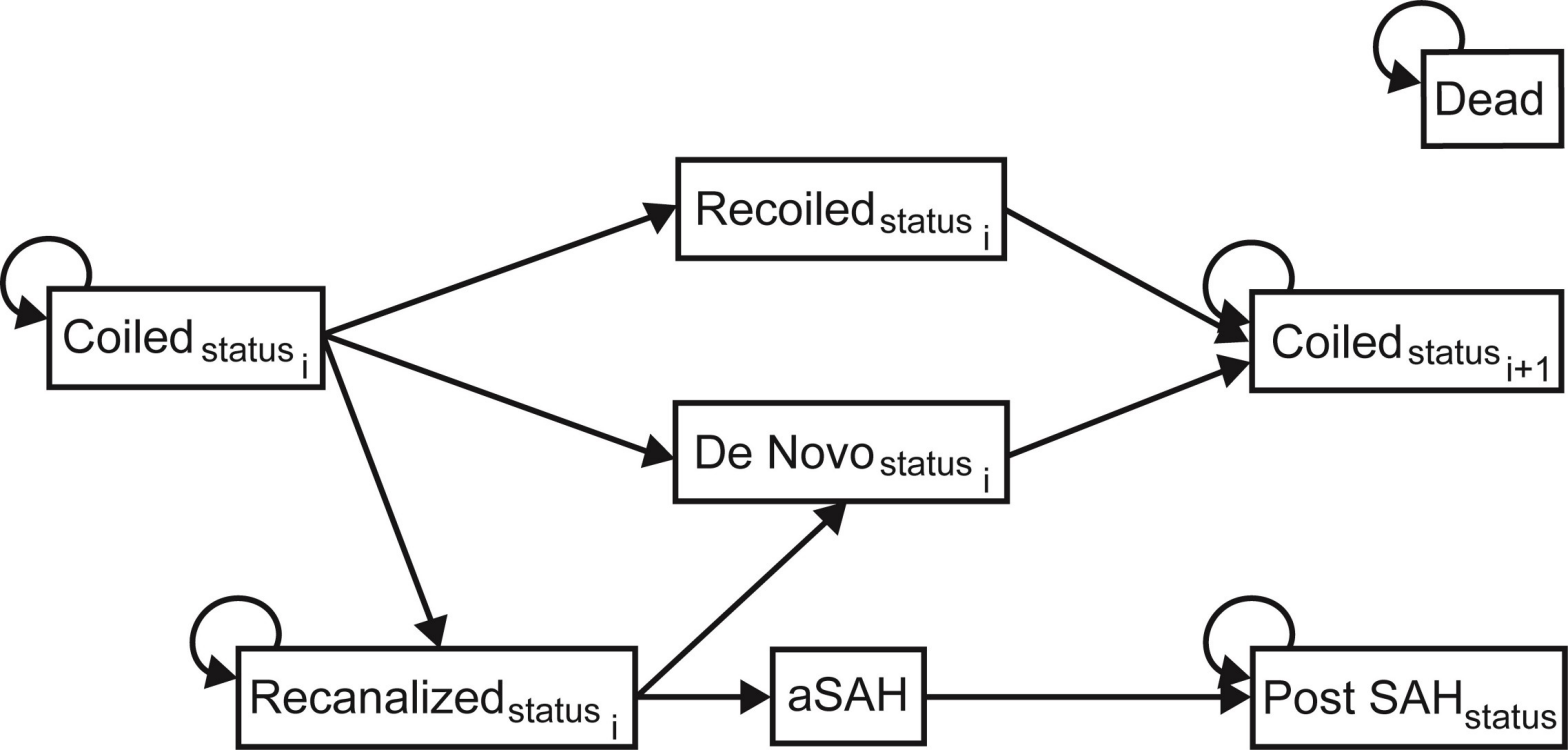
Markov model diagram illustrating all health states and possible transitions. All states may transition to *Dead*, however, these arrows have been left out for visual simplicity. Abbreviations: SAH–subarachnoid hemorrhage; aSAH–aneurysmal subarachnoid hemorrhage; mRS–modified Rankin Score; *status*–can be good function (mRS = 0), mild disability (mRS = 1–2), or moderate to severe disability (mRS = 3–5); *i*–can be 1 to 4 and represents the total number of coiling procedures a patient has had at any given time.

All patients entered the model at age 45 after undergoing coil embolization for their UIA. Age 45 was chosen as patients present with UIA most frequently in their 4^th^ and 5^th^ decade [5]. Patients were divided into two cohorts, one that received standard treatment, and one that received standard treatment plus the HTI adjunct. This HTI was only administered during the index coiling procedure. The model was constructed in Microsoft Excel^®^ (Version 16) and all Monte Carlo simulations were coded in Visual Basic^®^ (Version 7). An illustration of the states and possible transitions in the model are summarized in Fig 1. A more detailed model diagram can be found in S2.01 Fig in S1 File.

### Model parameters

The transitional probabilities as well as all the costs and utilities associated with each state were derived from the published scientific literature and are summarized in Table 1. An extensive MEDLINE search was performed to identify publications reporting each variable and preference was given to those publications with higher quality evidence, larger sample size, and more recent publication date. Once the parameters were selected, they were presented to vascular neurosurgery and interventional neuroradiology experts (APM, ME) and assessed for face validity. Complete details of the MEDLINE searches and model parameter derivations can be found in Supplement 3 in S1 File.

**Table 1:** Summary of the Markov model parameter values and their respective probabilistic distributions.

#	Variable	Value^*^ (events/N)		SE^*^ (Dist’n)	Sensitivity Analysis^†^		Study Design
					*Lower Value (SD)*	*Upper Value (SD)*	
**Transitional Probabilities**							
1	Probability of developing mild disability (mRS = 1–2)	0.048 (35/730) per procedure		0.005944 (Beta)	0.0361	0.0599	Meta-Analysis [24]
2	Probability of developing moderate to severe disability (mRS = 3–5)	0.022 (16/730) per procedure		0.002730 (Beta)	0.0165	0.0275	Meta-Analysis [24]
3	Probability of death from a coiling procedure	0.02 (59/5044) per procedure		0.002136 (Beta)	0.0157	0.0243	Meta-Analysis [6]
4	Probability of developing a recanalization	0.244 (321/1316) per 6 years		0.03592 (Beta)	0.1722	0.3158	Meta-Analysis [6] Retrospective Cohort [25]
5	Probability of coiled aneurysm re-treatment	0.091 (166/1699) per 6 years		0.01340 (Beta)	0.0642	0.1178	Meta-Analysis [6] Retrospective Cohort [25]
6	Probability of developing a de novo aneurysm	0.006 (62/2219) per year		0.001352 (Beta)	0.00330	0.00870	Meta-Analysis [26]
7	Probability of having an aSAH with an untreated aneurysm	0.014 (230/8382) per year		0.001275 (Beta)	0.0114	0.0166	Meta-Analysis [27]
8	Probability of death from an aSAH prior to reaching hospital	0.124 (578/3832) per event		0.007653 (Beta)	0.1087	0.1393	Meta-Analysis [28]
9	Probability of death from an aSAH after reaching hospital	0.265 (12797/48389) per event		0.002005 (Beta)	0.2610	0.2690	Retrospective Cohort [29]
10	Probability of developing mild disability (mRS = 1–2) from aSAH	0.2665 (12896/48389) per event		0.002010 (Beta)	0.26248	0.27052	Retrospective Cohort [29]
							Systematic Review [30]
11	Probability of developing moderate to severe disability (mRS = 3–5) from aSAH	0.1475 (7137/48389) per event		0.001612 (Beta)	0.14428	0.15072	Retrospective Cohort [29]
							Systematic Review [30]
**Costs**							
12	Coiling without complications	$30,013 per procedure		15905 (TNormal-O)	0^‡^ (0)	$46,473^‡^ (13,484)	Retrospective Cohort [31]
13	Coiling with complications leading to functional disability	$47,237 per procedure		22306 (TNormal-O)	$30,889^‡^ (14,861)	$83,016^‡^ (14,870)	Retrospective Cohort [31]
14	Coiling procedure with complications leading to death	$65,336 per procedure		58090 (TNormal-O)	$48,688^‡^ (16,756)	$181,515	Retrospective Cohort [31]
15	Care of coiled patient with good function (mRS = 0)	$11,197 per year		2799 (TNormal-O)	$5,598	$12,072^‡^ (2,694)	RCT [32]
16	Care of coiled patient with mild disability (mRS = 1–2)	$12,132 per year		3033 (TNormal-O)	$11,259^‡^ (2,703)	$18,198^‡^ (30)	RCT [32]
17	Care of coiled patient with moderate to severe disability (mRS = 3–5)	$42,257 per year		10564 (TNormal-O)	$21,128	$63,385	RCT [32]
18	Hospital care for aSAH	$93,440 per event		539 (Gamma)	$92,362	$94,517	Retrospective Cohort [29]
**Utilities**							
19	Coiled with good functional status (mRS = 0)	45–54	0.87	0.01 for all (TNormal-O)	0.840	0.880	Prospective Cross-Sectional [33]
		55–64	0.85				
		65–74	0.86				
		≥ 75	0.84				
20	Coiled with mild disability (mRS = 1–2)	0.72		0.025 (TNormal-O)	0.670	0.770	Systematic Review [34]
21	Coiled with moderate to severe disability (mRS = 3–5)	0.41		0.085 (TNormal-O)	0.240	0.580	Systematic Review [34]
22	Having an untreated aneurysm	-0.07		0.04082 (Beta)	0	-0.152	Prospective Cohort [35]
23	aSAH	0.41		0.085 (TNormal-O)	0.240	0.580	Systematic Review [34]
**Health Technology Innovation**							
24	HTI Efficacy	0 to 100% relative risk reduction		uniform	NA	NA	Model Assumption
25	Cost of HTI	$10,000 per procedure		Deterministic	NA	NA	Narrative Review [36]

**Note:** All currency represented in 2018 United States dollars. Abbreviations: N–total number of observations; SE–standard error; Dist’n–distribution; SD–standard deviation; mRS–modified Rankin Score; HTI–health technology innovation; TNormal-O–truncated ordered normal distribution; RCT–randomised controlled trial.

^*^A detailed description of how these variables and distributions were derived is available in Supplement 3 in S1 File.

^†^Values of two SE below and above the mean were used in the sensitivity analysis, unless indicated by a

“‡” (for more details please see Supplement 5 in S1 File).

This model can be used to represent any HTI that is administered during the index coiling procedure and acts to reduce the recanalization rate. In its simplest form, the HTI can be thought of as an improved coil that has a decreased incidence of aneurysm recanalization. We have included the use of mesenchymal stem cell therapy for the base case to demonstrate the application of this model. While mesenchymal stem cell therapy for UIA is under active investigation, its efficacy is still theoretical. Therefore, we chose to estimate the recanalization relative risk reduction (RRR) from a uniform distribution bounded by 0% and 100%. This assumes in the base case an expected RRR of 50%, lowering the absolute recanalization rate from 24.4% to 12.2%. In more advanced simulations, we vary the expected efficacy across all RRR values, thereby eliminating this assumption from the model.

### Assumptions

A detailed description of the model’s major assumptions and justifications can be found in Supplement 4 in S1 File.

### Base case

Using an iteration simulation, we calculated that 7,000 iterations were needed to generate a stable result (Supplement 5, S5.01 Fig in S1 File). Once the 7,000 iterations were complete, we calculated the incremental cost effectiveness ratio (ICER), the cost-effectiveness acceptability curve (CEAC), and the expected value of perfect information (EVPI) using standard health economic approaches [37]. This Monte Carlo simulation with 7,000 iterations was repeated 100 times, allowing us to determine both the expected value as well as the credible interval (CrI) for each result. A probabilistic one-way sensitivity analysis was then performed. Detailed explanations of these calculations, as well as all subsequent simulations, are available in Supplement 5 in S1 File.

### HTI cost-elasticity

To estimate how the cost of the HTI and its efficacy (the RRR of aneurysm recanalization) influence cost-effectiveness, we ran a ‘HTI cost-elasticity calculation’. This calculation determines the maximum price a healthcare payer would be willing to spend for the HTI with a given efficacy and threshold. The threshold is used to define the healthcare payer’s maximum willingness-to-pay for an increase of 1 QALY. All values of the RRR in aneurysm recanalization from 0% to 100% (in increments of 1%) were tested at thresholds of $50,000, $100,000, and $150,000 per QALY.

### Scenario analyses

We repeated the base case using three additional distributions for the RRR of aneurysm recanalization. All of these were normal distributions bounded by 0 and 1, with means (and standard deviations (SD)) of 10% (2%), 30% (5%), and 50% (15%).

### EVPPI

Using an expected value of partial perfect information (EVPPI) simulation, we calculated the expected benefit of resolving the uncertainty associated with patients’ baseline risk of aneurysm recanalization. In this simulation, we drew the HTI aneurysm recanalization RRR from a normal distribution bounded by 0 and 1 with a mean (SD) of 50% (15%). As in the EVPI simulations, we estimated the number of UIA coiled per year in the USA to be 15,925 [5] and assumed the HTI therapy would be used for 10 years.

## Results

### Base case

The base case analysis results are summarized in Table 2. The CEAC can be found in S5.02 Fig in S1 File and shows how the probability of cost-effectiveness when using mesenchymal stem cell therapy varies with changes in the willingness-to-pay threshold. Additionally, S5.03 Fig in S1 File illustrates how the EVPI varies with changes in the threshold.

**Table 2:** Probabilistic Monte Carlo simulation results for base case and scenario analyses.

Distribution for HTI Efficacy		Total Average Discounted Costs* ($ [95% CrI])		Total Average Discounted QALY* [95% CrI]		ICER ($ per QALY [95% CrI])	Probability MSC Therapy is Cost-Effective at Thresholds of:			10-Year EVPI ($ [95% CrI]) at Thresholds of:		
		*MSC Therapy*	*Standard Therapy*	*MSC Therapy*	*Standard Therapy*		*$50*,*000 per QALY*	*$100*,*000 per QALY*	*$150*,*000 per QALY*	*$50*,*000 per QALY*	*$100*,*000 per QALY*	$150,000 per QALY
**Base Case**		320,673 [191,700, 450,238]	313,490 [184,475, 443,093]	18.282 [17.923, 18.643]	18.130 [17.780, 18.483]	47,352 [46,337, 48,366]	0.485 [0.473, 0.496]	0.692 [0.680, 0.704]	0.780 [0.771, 0.790]	443,278,460 [423,078,518, 463,478,403]	228,663,907 [219,732,823, 238,594,990]	161,246,127 [127,726,195, 194,766,059]
**Scenario Analyses Using Normal Distributions of μ (σ):**	*10 (2)*	323,029 [195,643, 453,604]	313,562 [186,201, 444,235]	18.159 [18.154, 18.163]	18.130 [17.801, 18.500]	329,246 [326,2, 332,489]	0 [0]	0.0003 [-0.0001, 0.0006]	0.010 [0.008, 0.013]	0 [0]	26,748 [–26,454, 79,951]	1,879,026 [1,184,196, 2,573,856]
	*30 (5)*	321,826 [193,443, 451,523]	313,459 [185,190, 443,435]	18.218 [17.882, 18.537]	18.130 [17.762, 18.475]	95,160 [94,246, 96,075]	0.030 [0.026, 0.036]	0.488 [0.477, 0.499]	0.859 [0.850, 0.868]	443,278,460 [423,078,518, 463,478,403]	228,663,907 [218,732,823, 238,594,990]	161,246,127 [127,726,195 194,766,059]
	*50 (15)*	320,715 [193,000, 450,858]	313,499 [185,953, 444,121]	18.280 [17.949, 18.602]	18.130 [17.762, 18.481]	48,113 [47,493, 48,734]	0.476 [0.466, 0.487]	0.875 [0.868, 0.882]	0.960 [0.955, 0.965]	255,353,340 [245,241,653, 265,465,26]	43,253,287 [39,517,770, 46,988,804]	14,369,496 [10,554,347, 18,184,645]

**Note:** All currency represented in 2018 United States dollars.

Abbreviations: HTI–health technology innovation; CrI–credible interval; MSC–mesenchymal stem cell; QALY–quality-adjusted life-years; ICER–incremental cost-effectiveness ratio; EVPI–expected value of perfect information; μ –mean; σ –standard deviation.

^*^Values derived by following a single patient through the model for 30 years, beginning at age 45.

### HTI cost-elasticity

**Fig 2:**
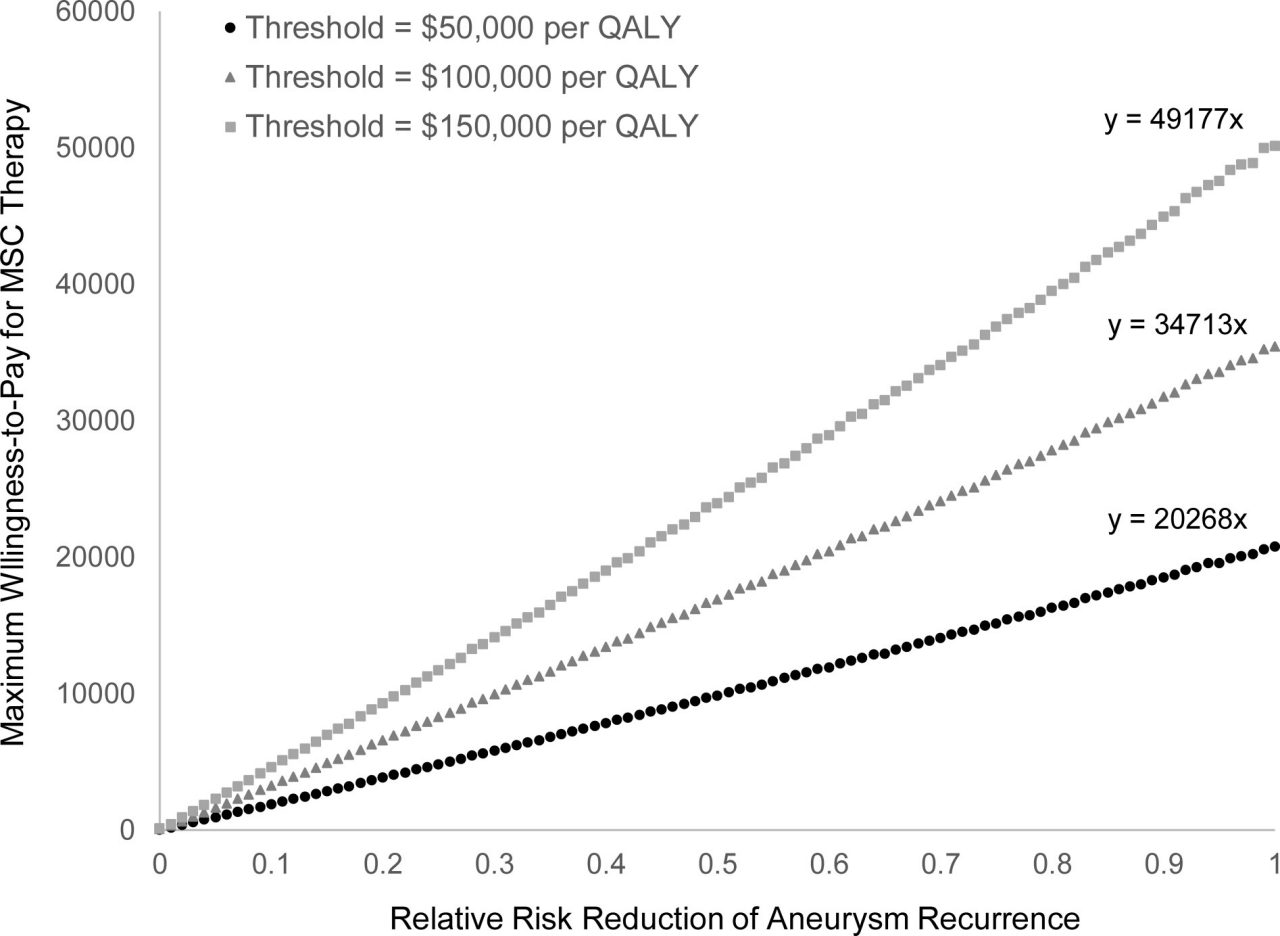
HTI cost-elasticity curve: How changes in HTI efficacy influence the maximum cost-effective price at various thresholds. Note: All currency represented in 2018 United States dollars. Equations can be used to calculate *y* (the maximum price the healthcare payer willing to pay for the HTI) for a given efficacy (*x*). Alternatively, equations can be rearranged to calculate *x*, minimum relative risk reduction in aneurysm recanalization the HTI must achieve in order to be cost-effective for a given price (*y*). Abbreviations: HTI–health technology innovation; MSC–mesenchymal stem cell; QALY–quality-adjusted life-years.

Fig 2 represents the main result of this paper and illustrates how varying the cost and efficacy of the HTI affects its cost-effectiveness at thresholds of $50,000, $100,000, and $150,000 per QALY. For a given aneurysm recanalization RRR (x-axis), the y-axis indicates what the maximum price a healthcare payer would be willing to pay for the novel therapy, while keeping the probability of cost-effectiveness greater than or equal to 0.5. For a given HTI cost (y-axis), the x-axis indicates the minimum RRR the therapy must provide in order for the probability of cost-effectiveness to be greater than or equal to 0.5.

### Healthcare payer perspective

Using Fig 2, healthcare payers can calculate the most they would be willing to spend on a HTI, given an expected RRR. For example, if a HTI imparts an aneurysm recanalization RRR of 30% (as in our scenario analysis), healthcare payers would be willing to spend at most $6,080 per procedure at a threshold of $50,000 per QALY (*y* = 20268 ∙ 0.30).

### Industry perspective

Using Fig 2, health technology innovators can calculate their expected yearly revenue, given a HTI efficacy. For example, if the expected RRR is 50% (as in our base case), healthcare payers would be willing to spend $9,849 per procedure at a threshold of $50,000 per QALY (*y* = 20268 ∙ 0.50). Assuming 15,925 UIA coiling procedures are performed each year [5], if all used the HTI, expected gross revenue would be $161,235,127 per year.

### Sensitivity analysis

**Fig 3:**
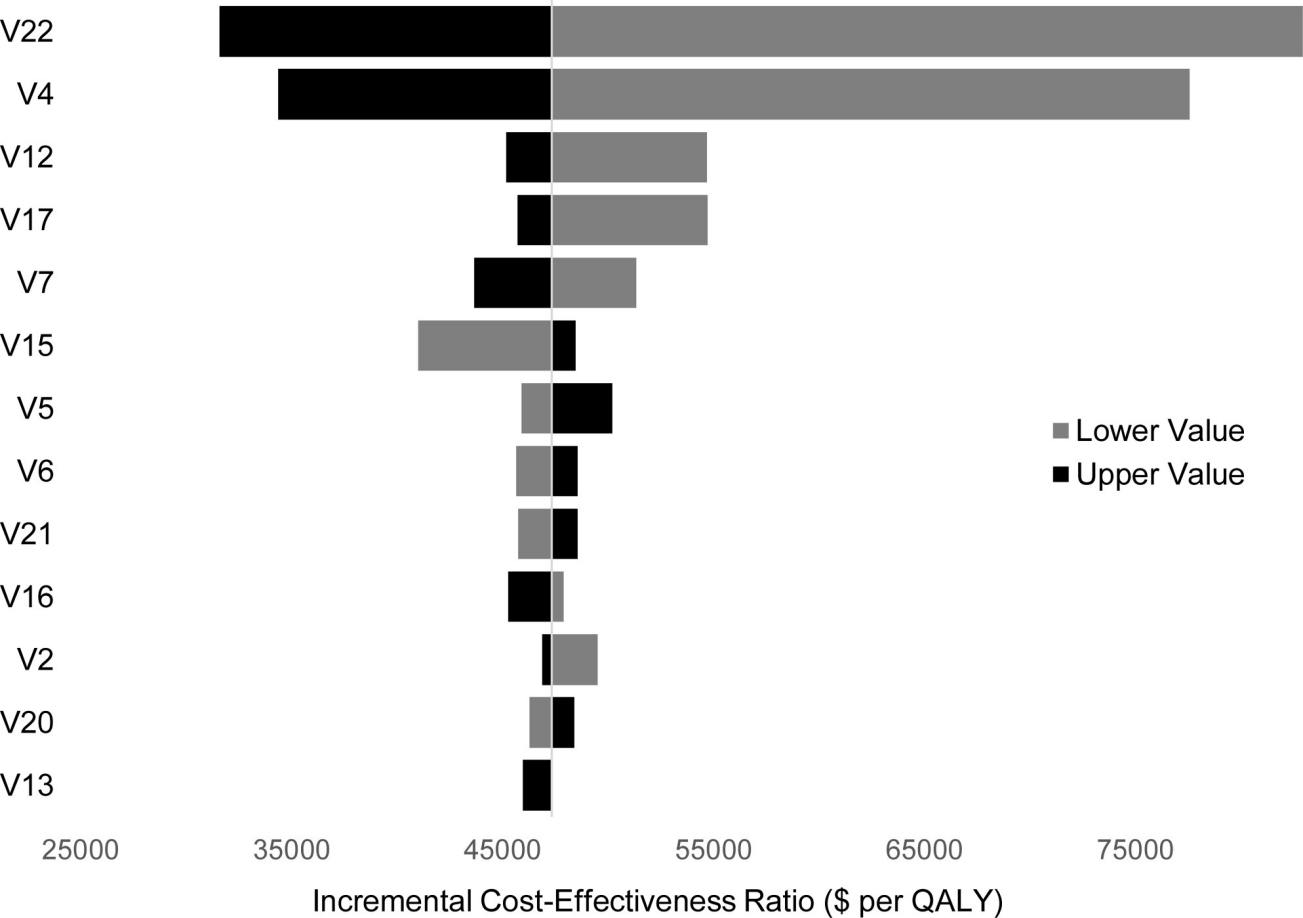
Tornado diagram for base case probabilistic sensitivity analysis. Note: All currency represented in 2018 United States dollars. Abbreviations: V–Variable Number (as defined in Table 1); QALY–quality-adjusted life-years.

The results of the one-way probabilistic sensitivity analysis are depicted in a tornado diagram Fig 3. The resulting ICERs with discount rates of 0%, 3%, and 5% were $34,493, $61,112, and $86,031 per QALY respectively. All variables absent from the tornado diagram had results that fell within the 95% CrI of the ICER in the base case. The variables with the most influence on the outcome of the model (ICER) were the utility lost from having a recanalized aneurysm that is not treated (Variable 22), the baseline probability of developing an aneurysm recanalization (Variable 4), and the healthcare costs associated with caring for a patient with moderate to severe disability (Variable 17). In all cases, increasing the given variable decreased the ICER, making the use of the HTI more likely to be cost-effective.

### Scenario analyses

The results of the scenario analyses are summarized in Table 2. In all of these simulations, the cost of the HTI (Variable 25) remained at $10,000 per procedure.

### EVPPI

The 10-year projected EVPPI for resolving the uncertainty surrounding the baseline rate of aneurysm recanalization (Variable 4) for thresholds of $50,000, $100,000, and $150,000 per QALY were calculated to be $113,336,994, $128,271, and $0 respectively.

## Discussion

In this study, we have built an economic model that is able to illustrate the costs and benefits of a new HTI that reduces the recanalization rate of coiled UIA. We provide numerical results for an illustrative base case, as well as demonstrate how changes in price, efficacy, and threshold affect the HTI’s cost-effectiveness. The application of this model is generalizable and can be used to assess any future HTI in this field. Researchers can populate the model with their preliminary efficacy and cost data in order to justify investment in larger animal or human trials. Once robust clinical data is available, all the described calculations can be repeated with the subsequent results tailored to a specific HTI, ultimately guiding healthcare payer funding decisions.

To our knowledge, this is the first cost-effectiveness analysis exploring the use of a HTI to reduce the recanalization rate of coiled UIA. There are previously developed economic models exploring the cost-effectiveness of coiling versus clipping of UIA; however, these studies focus on determining whether UIA should be treated [7, 9, 10, 38]. Our study focuses specifically on patients with UIA who will undergo endovascular coiling and asks whether they would benefit from a HTI coiling adjunct. Therefore, these previous models could not be applied to our research question, necessitating the development of a new model. Furthermore, a major improvement of our model is the inclusion of repeat aneurysm coiling procedures, which has been absent from all previous analyses. This is a significant advantage given the retreatment rate for coiled UIA is approximately 9% and represents a substantial added cost that is not incurred when aneurysms are treated by surgical clipping [6].

The main result of this study is summarized in Fig 2 which illustrates the maximum amount of money a healthcare payer is willing to spend for a HTI, based on an expected aneurysm recanalization RRR and a given threshold. Any combination of RRR and HTI price that falls below the chosen threshold line is considered cost-effective. In the early stages of HTI development, when preliminary clinical efficacy data is available, researchers and industry can use this figure to estimate (based on an expected RRR) the maximum price they can likely charge and predict the feasibility of pursuing further development. Once these technologies are developed, healthcare payers can use Fig 2 to calculate the amount they would be willing to spend on the HTI, maintaining a cost-effective healthcare system.

Our one-way sensitivity analysis (Fig 3) showed that increases and decreases of each variable had the expected directional impact on the ICER, supporting the internal validity of the model. The sensitivity analysis also highlights the variables which have the greatest impact on our model outcomes. The tornado diagram shows that the utility lost from having an unprotected aneurysm (Variable 22) has a significant effect on whether the HTI is cost-effective, consistent with the findings of Greving et al. [7]. If the utility lost is sufficiently low, the HTI would no longer be cost-effective. While we acknowledge the low quality of evidence surrounding this variable, anecdotal evidence suggests that patients do experience a decrease in their quality of life from the knowledge of having an unprotected aneurysm. Currently, a utility loss of 0.07 [35] is the best evidence available.

Another variable with significant impact on HTI cost-effectiveness is the probability of aneurysm recanalization (Variable 4). With higher recanalization rates, healthcare payers would be willing to spend more on the HTI, whereas with lower recanalization rates, the HTI is no longer cost-effective. The volatility of the ICER with changes in baseline aneurysm recanalization rates suggests that a strategy where only high-risk patients receive the HTI may be more beneficial. This is similar to the approach taken in stratifying rupture-risk in patients with UIA to determine who may benefit most from treatment. Using an EVPPI calculation, we measured the additional expected benefit to the healthcare system if we could perfectly predict a patient’s recanalization rate and only offer the HTI when cost-effective. Our results showed a 10-year EVPPI of $113,336,994 using a threshold of $50,000 per QALY. This suggests that there is considerable value in identifying patients at higher risk of recanalization. Thus, if a HTI is developed in the future, aneurysm recanalization risk stratification represents a critical area of research.

### Limitations

The primary limitation of this study stems from the paucity of clinical knowledge regarding HTI efficacy. Although we attempted to test all possible efficacy levels, some simulations including the base case required a specified RRR value and distribution; therefore, our results need to be interpreted with this consideration. While this stands as a current limitation, the constructed model can be easily adjusted once clinical efficacy data is available, extending its application into the future. Another limitation involves the model’s administration of the HTI exclusively at the index procedure. This allowed our results to reflect a direct comparison between standard treatment and standard treatment plus the HTI adjunct for a single coiling procedure but ignored the possibility of repeat HTI administration in subsequent procedures. In future, we can use our model to test an alternative strategy, for instance one in which all coiling procedures utilize the HTI, permitting we have the necessary efficacy data. Additionally, we acknowledge that this model assumes no difference in post-coiling management between treatment groups. If the HTI improves recanalization rates, it is likely that there would be an overall decrease in post-coiling management, including follow-up and imaging studies, which would decrease costs. Not accounting for this difference lead to more conservative results, underestimating the benefits of the HTI. Changes in follow-up management can be incorporated within the model, provided that evidence for changes in practice patterns exist. Finally, the model was designed specifically to be used by academia, industry, and government to guide investment in HTI and guide cost-effective pricing. Therefore, the model was not built from the societal perspective, and ignores all costs not covered by the healthcare system. Once HTI efficacy data is available, a societal cost scenario analysis is needed to properly reflect the impact the HTI will have on society at large.

## Conclusion

This economic analysis reveals several reasonable combinations of HTI price and recanalization RRR which are cost-effective at a threshold of $50,000 per QALY, suggesting that cost-effectiveness should not be a barrier to innovation. These results suggest that through HTI, a significant opportunity exists to improve the quality of life of patients with UIA while making appropriate use of scarce healthcare resources. In the future, our study can be applied to determine whether a HTI is worth developing, as well as how it should be priced. When a specific HTI reaches clinical application, our results also indicate that stratification by predicting aneurysm recanalization risk will be a critical area of research. We hope this economic analysis spurs government, industry, and academia toward further innovation in this field.

## Supporting information

S1 File Supplementary material parts 1 to 5. (PDF)
