# Supplementary material for: Innovation in unruptured intracranial aneurysm coiling: At which price or efficacy are new technologies cost-effective?
Source: PLoS One. 2021 Aug 9;16(8):e0255870. doi: 10.1371/journal.pone.0255870 (PMC8351982; doi:10.1371/journal.pone.0255870)
Supplement: S1 File — (PDF) [file pone.0255870.s001.pdf]

Supplemental Material

Supplement 1

Table S1.01. Reporting checklist from the Consolidated Health Economic Evaluation Reporting Standards statement 2
Table S1.02. Reporting checklist from the Second Panel on Cost-Effectiveness in Health and Medicine 5

Supplement 2

Fig S2.01. Detailed Markov Model Diagram Illustrating All Health States and Possible Transitions 7
Description of Markov model structure 7

Supplement 3

Derivation of the model parameters and their distributions 9

Supplement 4

Model assumptions and their respective justifications 18

Supplement 5

Model simulation methodology 23
Optimizing number of iterations per simulation 23
Fig S5.01. Determination of optimal number of iterations for probabilistic Monte Carlo simulation 24
Base case 24
CEAC 24
Fig S5.02. Cost-effectiveness acceptability curve 25
EVPI 25
Fig S5.03. EVPI over threshold 27
Sensitivity analysis 27
HTI cost-elasticity 28
Scenario analyses 29
EVPPI 29

References 31

Supplement 1

Table S1.01. Reporting checklist from the Consolidated Health Economic Evaluation Reporting Standards statement [1]

Section/Item	Item No.	Recommendation	Reported on page
**Title and abstract**			
Title	1	Identify the study as an economic evaluation or use more specific terms such as "cost-effectiveness analysis", and describe the interventions compared.	i
Abstract	2	Provide a structured summary of objectives, perspective, setting, methods (including study design and inputs), results (including base case and uncertainty analyses), and conclusions.	1
**Introduction**			
Background and objectives	3	Provide an explicit statement of the broader context for the study.	3-4
		Present the study question and its relevance for health policy or practice decisions.	4
**Methods**			
Target population and subgroups	4	Describe characteristics of the base case population and subgroups analyzed, including why they were chosen.	4-5
Setting and location	5	State relevant aspects of the system(s) in which the decision(s) need(s) to be made.	4-5
Study perspective	6	Describe the perspective of the study and relate this to the costs being evaluated.	4-5
Comparators	7	Describe the interventions or strategies being compared and state why they were chosen.	4-5
Time horizon	8	State the time horizon(s) over which costs and consequences are being evaluated and say why appropriate.	4-5, Supp. 15
Discount rate	9	Report the choice of discount rate(s) used for costs and outcomes and say why appropriate.	4
Choice of health outcomes	10	Describe what outcomes were used as the measure(s) of benefit in the evaluation and their relevance for the type of analysis performed.	5
Measurement of effectiveness	11a	*Single study-based estimates:* Describe fully the design features of the single effectiveness study and why the single study was a sufficient source of clinical effectiveness data.	NA
	11b	*Synthesis-based estimates*: Describe fully the methods used for identification of included studies and synthesis of clinical effectiveness data.	5, Supp. 9-14
Measurement and valuation of preference based outcomes	12	If applicable, describe the population and methods used to elicit preferences for outcomes.	5, Supp. 9-14
Estimating resources and costs	13a	*Single study-based economic evaluation:* Describe approaches used to estimate resource use associated with the alternative interventions. Describe primary or secondary research methods for valuing each resource item in terms of its unit cost. Describe any adjustments made to approximate to opportunity costs.	NA

Section/Item	Item No.	Recommendation	Reported on page
**Methods (continued)**			
Estimating resources and costs (continued)	13b	*Model-based economic evaluation:* Describe approaches and data sources used to estimate resource use associated with model health states. Describe primary or secondary research methods for valuing each resource item in terms of its unit cost. Describe any adjustments made to approximate to opportunity costs.	5-6, Supp. 9-14
Currency, price date, and conversion	14	Report the dates of the estimated resource quantities and unit costs. Describe methods for adjusting estimated unit costs to the year of reported costs if necessary. Describe methods for converting costs into a common currency base and the exchange rate.	Supp. 9-14
Choice of model	15	Describe and give reasons for the specific type of decision-analytical model used. Providing a figure to show model structure is strongly recommended.	4
Assumptions	16	Describe all structural or other assumptions underpinning the decision-analytical model.	Supp. 7-8, Supp. 15-17
Analytical methods	17	Describe all analytical methods supporting the evaluation. This could include methods for dealing with skewed, missing, or censored data; extrapolation methods; methods for pooling data; approaches to validate or make adjustments (such as half cycle corrections) to a model; and methods for handling population heterogeneity and uncertainty.	6-8 Supp. 18-23
**Results**			
Study parameters	18	Report the values, ranges, references, and, if used, probability distributions for all parameters. Report reasons or sources for distributions used to represent uncertainty where appropriate. Providing a table to show the input values is strongly recommended.	8-10, 22-24, Supp. 9-14
Incremental costs and outcomes	19	For each intervention, report mean values for the main categories of estimated costs and outcomes of interest, as well as mean differences between the comparator groups. If applicable, report incremental cost-effectiveness ratios.	25
Characterizing uncertainty	20a	*Single study-based economic evaluation:* Describe the effects of sampling uncertainty for the estimated incremental cost and incremental effectiveness parameters, together with the impact of methodological assumptions (such as discount rate, study perspective).	NA
	20b	*Model-based economic evaluation:* Describe the effects on the results of uncertainty for all input parameters, and uncertainty related to the structure of the model and assumptions.	8-10, 25
Characterizing heterogeneity	21	If applicable, report differences in costs, outcomes, or cost-effectiveness that can be explained by variations between subgroups of patients with different baseline characteristics or other observed variability in effects that are not reducible by more information.	NA
**Discussion**			
Study findings, limitations, generalizability, and current knowledge	22	Summarize key study findings and describe how they support the conclusions reached. Discuss limitations and the generalizability of the findings and how the findings fit with current knowledge.	10-13

Section/Item	Item No.	Recommendation	Reported on page
**Other**			
Source of funding	23	Describe how the study was funded and the role of the funder in the identification, design, conduct, and reporting of the analysis. Describe other non-monetary sources of support.	14
Conflicts of interest	24	Describe any potential for conflict of interest of study contributors in accordance with journal policy. In the absence of a journal policy, we recommend authors comply with International Committee of Medical Journal Editors recommendations.	14

Table S1.02. Reporting Checklist from the Second Panel on Cost-Effectiveness in Health and Medicine [2]

Element	Journal Article	Supplemental Material
**Introduction**		
Background of the problem	3-4	
**Study Design and Scope**		
Objectives	4	
Audience	4-5	
Type of analysis	4	
Target populations	5	
Description of interventions and comparators (if applicable)	5-6	
Other intervention descriptors (eg, care setting, model of delivery, intensity and timing of intervention)	NA	
Boundaries of the analysis; defining the scope or comprehensiveness of the study (eg, for a screening program, whether only a subset of many possible strategies are included; for interventions with many possible delivery settings, whether only one or more settings are modeled)	5-6	
Time horizon	5	
Analytic perspectives (eg, reference case perspectives [health care sector, societal]; other perspectives such as employer or payer)	4	
Whether this analysis meets the requirements of the reference case	5	
Analysis plan	6-8	
**Methods and Data**		
Trial-based analysis or model-based analysis. If model-based:		
Description of event pathway or model (describe condition and health states)	4-5	7-8
Diagram of event pathway or model (depicting the sequencing and possible transitions among the health states included)	21	7
Description of model used (eg, decision tree, state transition, microsimulation)	4	7-8
Modeling assumptions	6, 22-24	15-17
Software used	5	
Identification of key outcomes	6-8	
Complete information on sources of effectiveness data, cost data, and preference weights	22-24	9-14
Methods for obtaining estimates of effectiveness	5-6	
Methods for obtaining estimates of costs and preference weights	5-6	9-14
Critique of data quality	12-13	
Statement of costing year (ie, year to which all costs have been adjusted)	4	11-12
Statement of method used to adjust costs for inflation	4	11-12
Statement of type of currency	4	11-12
Source and methods for obtaining expert judgment if applicable	NA	
Statement of discount rates	4	

Element	Journal Article	Supplemental Material
**Impact Inventory**		
Full accounting of consequences within and outside the health care sector	NA	
**Results**		
Results of model validation	8-9, 25	
Reference case results (discounted and undiscounted): total costs and effectiveness, incremental costs and effectiveness, etc.	8, 19-20	18-22
Disaggregated results for important categories of costs, outcomes, or both	NA	
Results of sensitivity analysis	9	21-22
Other estimates of uncertainty	10, 25	19-21
Graphical representation of cost-effectiveness results	21	20-21
Graphical representation of uncertainty analyses	21	20-21
Aggregate cost and effectiveness information	21, 25	20-21
Secondary analyses	17, 25	
**Disclosures**		
Statement of any potential conflicts of interest due to funding source, collaborations, or outside interests	14	
**Discussion**		
Summary of reference case results	10	
Summary of sensitivity of results to assumptions and uncertainties	11-12	
Discussion of the study results in the context of related cost-effective analyses	10-11	
Discussion of ethical implications	NA	
Limitations of the study	13	
Relevance of study results to specific policy questions or decisions	10-11, 14	

Supplement 2

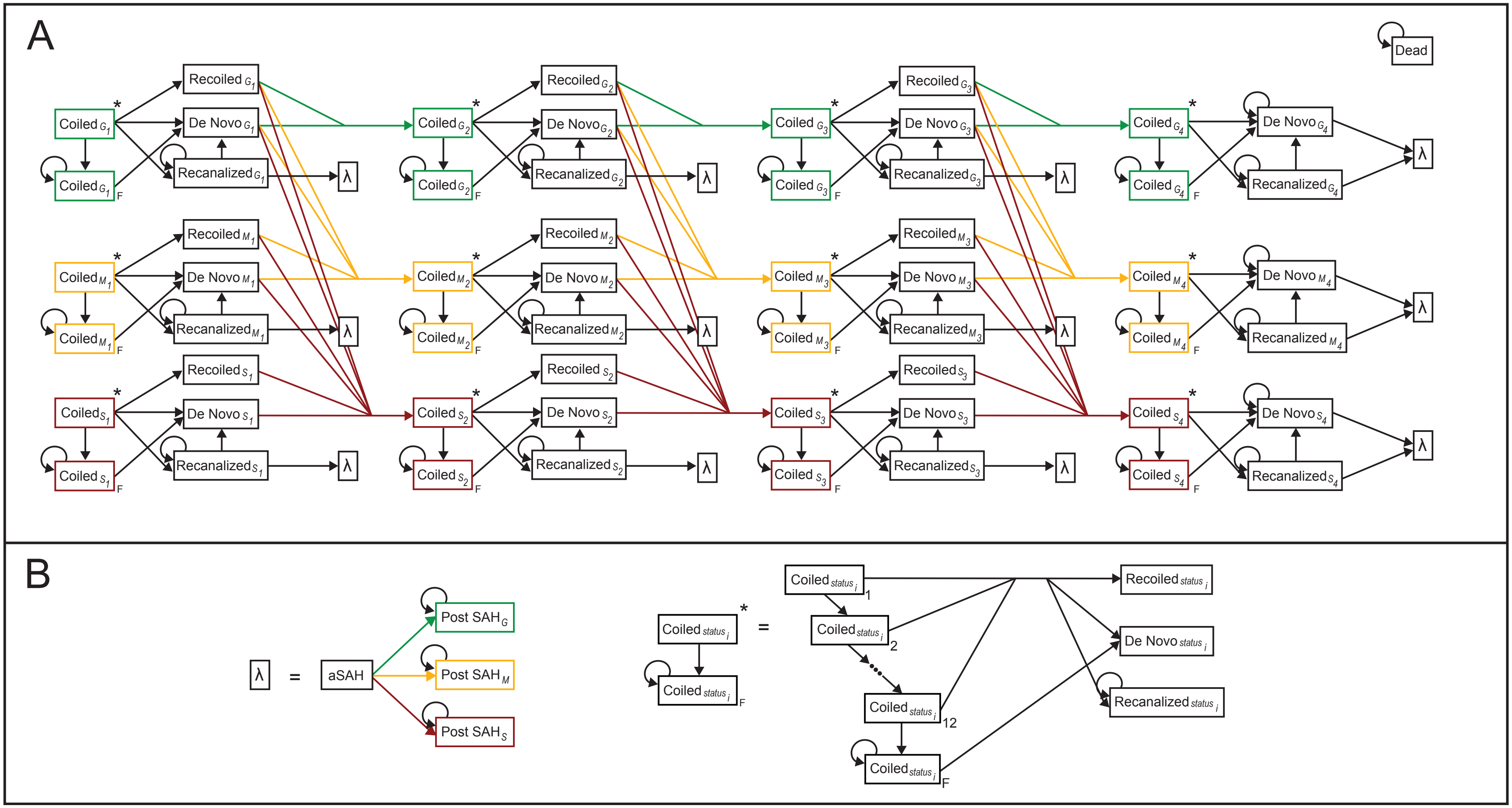

Fig S2.01. Detailed Markov model diagram illustrating all health states and possible transitions

A – Markov Model. B – Legend.

Note: All states may transition to *Dead*, however, these arrows have been left out for visual simplicity.

Abbreviations: SAH – subarachnoid hemorrhage; aSAH – aneurysmal subarachnoid hemorrhage; mRS – modified Rankin Score; G – good function (mRS = 0); M – mild disability (mRS = 1-2); S – moderate to severe disability (mRS = 3-5); *status* – can be G, M, or S; *i* – can be 1 to 4 and represents the total number of coiling procedures a patient has had at any given time.

Markov Model Structure

Following the index coiling procedure, patients enter the *Coiled* state where their aneurysm is assumed to be treated. During the coiling procedure, complications may not occur, leading to good post-operative function (defined as modified Rankin Score (mRS)=0), or complications may occur which lead to mild disability (defined as mRS=1-2), moderate to severe disability (defined as mRS=3-5), or death. Therefore, there is a separate *Coiled* state for each functional status (good, mild disability, and moderate to severe disability). While in *Coiled*, patients may remain in place or they may transition to one of three other states. Patients may develop a recanalization that is treated and transition to *Recoiled*, develop a recanalization that is not treated and transition to *Recanalized*, or develop a de novo aneurysm that is treated and transition to *De Novo*. These three states also depend on functional disability, where patients can only transition from *Coiled* to states with the same functional status. For example, a patient that is in $Coiled_{Mild}$ would transition to $Recoiled_{Mild}$ as opposed to $Recoiled_{Good}$. Patients may only transition to *Recoiled* or *Recanalized* within 6 years (12 cycles) of a coiling procedure. After this time, the rate of aneurysm recanalization drops to 0%, however, patients may still develop a de novo aneurysm.

From the $Recoiled_i$ and $De\ Novo_i$ states, after having their aneurysm (re)coiled, patients transition to a new *Coiled* state with the subscript $i+1$. However, as in the index procedure, on account of procedural complications, patients may again develop deficits and enter a state with lower functional status. In this model, patients may maintain or decline in functional status but cannot improve.

While in *Recanalized*, patients may remain in place, or develop a de novo aneurysm and transition to *De Novo*. Alternatively, since these patients have a recanalized unprotected aneurysm, although rare, they may develop an aneurysmal subarachnoid hemorrhage (aSAH). Following an aSAH, patients may die immediately and transition directly to *Dead*. Alternatively, patients transition to the *aSAH* state, and from there may die in hospital and transition to *Dead*, or may survive and transition to *Post SAH*. As in the case of coiling procedures, aSAH can cause a permanent decrease in a patient's functional status. Therefore, there is a separate state of *Post SAH* for each functional status (good, mild disability, and moderate to severe disability). Once in *Post SAH*, patients remain in this state for the remainder of their lives.

After patients transition from their fourth coiling procedure to $Coiled_4$, they are no longer offered aneurysmal treatment in this model (see Supplement 4, Assumption S4.05 for justification). Therefore, if they develop a de novo aneurysm or a recanalization, further treatment is not pursued. However, patients in both these states ($Recanalized_4$ and $De\ Novo_4$) will still be at risk of developing an aSAH, since they have an unprotected intracranial aneurysm.

Patients in this model may die as a result of a complication from an endovascular coiling procedure, an aSAH, or from non-aneurysmal causes. Rates for non-aneurysmal causes of death are based on age-specific population statistics [3]. Patients may therefore transition from any state to *Dead*, however, these arrows have been removed by convention from Fig 1 and S2.01 for visual simplicity.

Supplement 3

To identify the relevant articles used to derive the model parameters, a MEDLINE search was performed using the following search terms:

- Intracranial Aneurysms: exp Aneurysm/; exp Aneurysm, Ruptured/; exp Intracranial Aneurysm/; exp Endovascular Procedures/; exp Embolization, Therapeutic/; exp Intracranial Hemorrhages/; exp Subarachnoid Hemorrhage/; aneurysm.tw; endovascular coil*.tw; coil embolization.tw; subarachnoid hemorrhage.tw; intracranial hemorrhage*.tw; recanalization.tw
- Quality of Life: exp "Quality of Life"/; exp Quality-Adjusted Life Years/; quality of life.tw; quality adjusted life years.tw; QALY.tw; short form health survey.tw; sf-36.tw; sf-12.tw; sf-6.tw; euroqol.tw; eq-5d.tw; utility*.tw; HRQoL.tw
- Costs: exp Economics, Medical/; exp Economics, Hospital/; exp Health Care Costs/; exp Cost-Benefit Analysis/; exp "Costs and Cost Analysis"/; exp "Cost of Illness"/; economics; resource allocation; health care rationing; health econ*.tw; health care cost.tw; cost-benefit.tw; cost-analysis.tw

Preference was given to articles with a higher-level study design including meta-analyses and randomized controlled trials. More recently published articles were also preferred. Once parameters were derived, they were screened by two independent experts in neurosurgery and neurointerventional radiology (APM, ME) for face validity. The following are detailed derivations of the model parameters and their distributions.

Variable 1: Probability of developing mild disability (mRS=1-2) from a coiling procedure
Variable 2: Probability of developing moderate to severe disability (mRS=3-5) from a coiling procedure

These transitional probabilities were derived from the meta-analysis by Lanterna et al. [4]. This article found that 56 of 794 (7%, 95%CI: [5.3, 8.7]) patients developed permanent disability following coil embolization of an UIA. Of the 53 patients, 51 of them had severity of deficits reported: 35 (69%) were mildly disabled and 16 (31%) developed moderate to severe disability. Using these proportions, of the 7% with permanent disability, 4.8% ($7\% \times 0.69$) were mildly disabled and 2.2% ($7\% \times 0.31$) were moderately to severely disabled. To calculate each variable's standard error, the overall 95% confidence interval was converted to confidence intervals for each variable by multiplying the upper and lower bound by the corresponding proportion for each disability level. With these new confidence intervals, (95%CI: [3.64, 5.97] for mild disability and 95%CI: [1.66, 2.73] for moderate to severe disability) the standard errors for each disability level were derived using the following formula:

Equation S3.01:

$$SE = \frac{UB - LB}{3.92}$$

where UB and LB represent the upper and lower bound of the 95%CI respectively.

After converting from percentages to probabilities, this resulted in standard errors of 0.005944 and 0.002730 for mild disability and moderate to severe disability respectively.

Both variables were drawn from beta distributions, a common distribution used for probabilities in economic analyses, as it inherently bounds the probability between 0 and 1. Values for α and β for each beta distribution were calculated using the following formulas:

Equation S3.02:

$$\alpha = \frac{\mu^2(1-\mu)}{\sigma^2} - \mu$$

Equation S3.03:

$$\beta = \left(\frac{\mu(1-\mu)}{\sigma^2} - 1\right)(1-\mu)$$

where μ represents the deterministic mean and σ represents the SE for each variable.

Variable 3: Probability of death from a coiling procedure

This transitional probability was derived from the meta-analysis by Naggara et al. [5]. This article found that 59 of 5044 (1.2%) patients died while undergoing an endovascular coiling procedure. With a random-effect weighted average, this rate was found to be 2.0% (99%CI: [1.5, 2.6]). Using the 99%CI, the SE was derived with the following formula:

Equation S3.04:

$$SE = \frac{UB - LB}{5.15}$$

where UB and LB represent the upper and lower bound of the 99%CI respectively.

After converting from a percent to a probability, this resulted in a SE of 0.00213592. This variable was drawn from a beta distribution, a common distribution used for probabilities in economic analyses, as it inherently bounds the probability between 0 and 1. The values for α and β for the beta distribution were calculated using Equations S3.02 and S3.03.

Variable 4: Probability of developing a recanalization of a coiled aneurysm

Variable 5: Probability of a coiled aneurysm requiring re-treatment

These transitional probabilities were derived from the meta-analysis by Naggara et al. [5]. This article found that 321 of 1316 (24.4%) coiled UIA recanalized, and 166 of 1699 (9.1%, 99%CI: [6.2, 13.1]) coiled UIA required retreatment. Naggara et al defined recanalization as all aneurysms that recanalized, including those that were and were not retreated, and defined re-treated as the proportion of all coiled UIA that were re-treated. Therefore, within our Markov model, Variable 5 represents the probability of transitioning from *Coiled* to *Recoiled* and $Variable\ 5 - Variable\ 4$ represents the probability of transitioning from *Coiled* to *Recanalized*. Because the SE of Variable 4 was not provided, it was approximated by applying a linear transformation to the SE of Variable 5. First the SE of Variable 5 was calculated using Equation S3.04, and after converting from a percent to a probability was calculated to be 0.01339806. The SE for Variable 4 was then calculated using the following formula:

Equation S3.05:

$$SE_4 = \frac{Variable\ 4}{Variable\ 5} \times SE_5$$

where $Variable$ 4 and $Variable$ 5 represents the deterministic values of each variable.

The SE for Variable 4 was therefore estimated to be 0.03592446. These variables were drawn from beta distributions, a common distribution type used for probabilities in economic analyses, as it inherently bounds the probability between 0 and 1. The values for α and β for each beta distribution were calculated using Equations S3.02 and S3.03. Naggara et al., however, did not provide clear time intervals for when these recanalizations or retreatments occurred. Therefore, the retrospective cohort study by Ries et al. which looked at the long-term outcomes of coiled UIA, was used to estimate this time interval [6]. Ries et al. found recanalizations occurred between 0 and 77 months from the index coiling procedure. Since our cycle length was 6 months and the majority of recanalizations occurred early in the follow-up period, this interval was approximated at 72 months (12 cycles). The temporal distribution of the recanalizations was not provided and therefore assumed to be uniform. After 12 cycles, the recanalization rate was assumed to be 0. Once these variables were calculated, they were converted to a per-cycle probability using the following formula:

Equation S3.06:

$$P_{per\ cycle} = \left(1 + P_{per\ i\ cycles}\right)^{\frac{1}{i}} - 1$$

where P is the transitional probability and i is the timeframe in number of cycles for the original transitional probability.

Variable 6: Probability of developing a de novo aneurysm

This transitional probability was derived from the meta-analysis by Giordan et al. [7]. This article found that 62 of 2219 (0.6%, 95%CI: [0.35, 0.88]) patients per year with a history of an UIA developed a de novo aneurysm. Using the 95%CI, the SE was derived using Equation S3.01. After converting from a percent to a probability, this resulted in a SE of 0.002995513. This variable was drawn from a beta distribution, a common distribution used for probabilities in economic analyses, as it inherently bounds the probability between 0 and 1. The values for α and β for the beta distribution were calculated using Equations S3.02 and S3.03. Once this variable was calculated, it was converted into a per-cycle probability using Equation S3.06, where $i = 2$.

Variable 7: Probability of having an aSAH with an untreated aneurysm

This transitional probability was derived from the meta-analysis by Greving et al. [8]. This article found that 230 of 8382 patients suffered from a rupture of their known UIA, over 29,166 patient-years. This resulted in an annual rupture risk of 1.4% (95%CI: [1.1, 1.6]). Using the 95%CI, the SE was derived using Equation S3.01. After converting from a percent to a probability, this resulted in a SE of 0.001275. This variable was drawn from a beta distribution, a common distribution used for probabilities in economic analyses, as it inherently bounds the probability between 0 and 1. The values for α and β for the beta distribution were calculated using Equations S3.02 and S3.03. Once this variable was calculated, it was converted into a per-cycle probability using Equation S3.06, where $i = 2$.

Variable 8: Probability of death from an aSAH prior to reaching hospital

This transitional probability was derived from the meta-analysis by Huang et al. [9]. This article found that 578 of 3832 (12.4%, 95%CI: [11, 14]) patients died following an aSAH prior to reaching hospital. Using the 95%CI, the SE was derived using Equation S3.01. After converting from a percent to a probability, this resulted in a SE of 0.007653. This variable was drawn from a beta distribution, a common distribution used for probabilities in economic analyses, as it inherently bounds the probability between 0 and 1. The values for α and β for the beta distribution were calculated using Equations S3.02 and S3.03.

Variable 9: Probability of death from an aSAH after reaching hospital

This transitional probability was derived from a National Inpatient Sample (NIS) Registry retrospective cohort study by Qureshi et al. [10]. This article found that 12,797 of 48,389 (26.5%) patients who reached hospital alive following an aSAH died during their hospital admission. The SE for this variable was not provided and therefore calculated to be 0.002001, using method of moments and the following formula:

Equation S3.07

$$SE = \sqrt{\frac{\alpha\beta}{(\alpha+\beta)^2(\alpha+\beta+1)}}$$

where $\alpha = 12{,}797$ and $\beta = 35{,}592$.

This variable was drawn from a beta distribution, a common distribution used for probabilities in economic analyses, as it inherently bounds the probability between 0 and 1.

Variable 10: Probability of developing mild disability (mRS=1-2) from an aSAH after reaching hospital

Variable 11: Probability of developing moderate to severe disability (mRS=3-5) from an aSAH after reaching hospital

These transitional probabilities were derived from a NIS Registry retrospective cohort study by Qureshi et al. [10] in conjunction with a systematic review by Hop et al. [11]. Qureshi et al. found that 20,033 of 48,389 (41.4%) patients who reached hospital alive following an aSAH had some level of disability following discharge from hospital. Hop et al. found that 14.75% of patients are moderately to severely disabled. Therefore, 26.65% of patients that reach hospital alive following aSAH were assumed to be mildly disabled. Using these percentages, we then calculated based on the Qureshi data that of the 20,033 disabled patients, 12,896 were mildly disabled and 7,137 were moderately to severely disabled. Since Qureshi did not provide SEs, they were calculated to be 0.002282 and 0.002127 for Variables 10 and 11 respectively, using Equation S3.07, where $\alpha = 12{,}896$ and $\beta = 35{,}493$ for Variable 10, and $\alpha = 7{,}137$ and $\beta = 41{,}252$ for Variable 11. These variables were drawn from beta distributions, a common distribution type used for probabilities in economic analyses, as it inherently bounds the probability between 0 and 1.

Variable 12: Healthcare cost of a coiling procedure without complications

Variable 13: Healthcare cost of a coiling procedure with complications leading to functional disability

Variable 14: Healthcare cost of a coiling procedure with complications leading to patient death

These healthcare costs were derived from the NIS Registry retrospective cohort study by Brinjikji et al. [12]. This article found that the median hospital costs for a coiling procedure was: without complications \$25,734 (IQR: [17436, 35846]), with major complications causing disability (mRS=1-5) \$40,502 (IQR: [24289, 50108]), and with complications causing mortality \$56,020, all in 2008 USD. Since the IQR was not reported for Variable 14, and given the cost was not statistically significantly different than the cost of a clipping procedure with complications leading to patient death, this IQR: [32972,100211] was used instead. These costs and IQRs were converted to 2018 USD using the CCEMG – EPPI-Centre Cost Converter (Version 1.6) (16.63% increase) giving the following values: Variable 12: \$30,013.56 (IQR: [20335.61, 41807.19]), Variable 13: \$47,237.48 (IQR: [28328.26, 58440.96]), Variable 14: 65336.13 (IQR: [38455.24, 116876.09]) [13]. Since the IQRs were provided in place of the SEs, we estimated the SE using the following formula:

Equation S3.08:

$$SE \cong \frac{IQR}{1.35}$$

which assumes the distribution is normal.

Because we had to assume a normal distribution, we drew these variables from a truncated normal distribution, bounded by 0 and four times the SE. In order to ensure that Variable 12 remained the least expensive while Variable 14 remained the most expensive when converting to a probabilistic model, we follow a Dirichlet style approach. Variable 12 was drawn from a truncated normal distribution with a mean of \$30,013.56 and a SE of \$15,9043.87. Variable 13 was calculated by first drawing the difference between Variable 13 and 12 from a truncated normal distribution with a mean of \$17,223.92. The SE for this distribution was calculated by applying a linear transformation to Variable 13's SE using the following formula:

Equation S3.09:

$$SE_{dec} = \frac{\mu_i - \mu_{i-1}}{\mu_i} SE_i$$

where SE_{dec} is the SE of the decrement between Variable i and Variable $i-1$, μ_i is the mean for Variable i, and SE_i is the standard error for Variable i; $i = 13$.

This difference was then added to the probabilistic value for Variable 12, resulting in the value for Variable 13. The same technique was used when calculating Variable 14, using the difference between Variable 13 and 14 ($i = 14$).

Variable 15: Healthcare cost for a patient with a coiled aneurysm living with good functional status (mRS=0)
Variable 16: Healthcare cost for a patient with a coiled aneurysm living with mild disability (mRS=1-2)
Variable 17: Healthcare cost for a patient with a coiled aneurysm living with moderate to severe disability (mRS=3-5)

These healthcare costs were derived from a contemporary stroke cohort of 958 patients and calculated in the SWIFT-PRIME Trial costs analysis by Shireman et al. [14]. This article

reported annual healthcare costs by patients' 90-day mRS in 2015 USD. Therefore, for each variable, the cost was calculated by taking a relative weighted average of costs for the relevant mRS using the following formula:

Equation S3.10:

$$Cost_{Vi} = \sum_{k=mRS_{LB}}^{mRS_{UB}} \left(Cost_k \frac{N_k}{\sum_{j=mRS_{LB}}^{mRS_{UB}} N_j} \right)$$

where $Cost_{Vi}$ is the cost for Variable i, mRS_{LB} is the lowest mRS score possible as Variable i is defined, mRS_{UB} is the highest mRS score possible as Variable i is defined, $Cost_k$ is the annual healthcare cost for a patient with m$RS = k$, and N_k is the number of patients with m$RS = k$.

The costs calculated for Variables 15, 16, and 17 were therefore \$10,569.00, \$11,452.00, and \$39,887.44 respectively. These costs were then converted from 2015 to 2018 USD using the CCEMG – EPPI-Centre Cost Converter (Version 1.6) (5.94% increase) giving the following values: Variable 15: \$11,196.80, Variable 16: \$12,132.25, and Variable 17: \$42,256.76 [13]. Since Shireman et al. did not report any variance for these values, we estimated the SEs for each variable as 25% of its deterministic value. To preserve the relative order of most to least costly, truncated normal distributions using the method described for Variables 12 to 14 were followed. All values were divided by 2 when entered into the model to reflect the 6-month cycle length.

Variable 18: Healthcare cost of treating a patient with an aSAH

This healthcare cost was derived from a NIS Registry retrospective cohort study by Qureshi et al. [10]. This article found that among 48,389 patients treated for SAH, the average hospital costs were \$65,900 with a SE of \$380, reported in 2001 USD. This cost and SE were converted to 2018 USD using the CCEMG – EPPI-Centre Cost Converter (Version 1.6) (41.79% increase) resulting in \$93,439.61 and \$538.80 respectively [13]. This variable was drawn from a gamma distribution, which is commonly used for modeling costs in economic analyses. The values for α and β for each gamma distribution were calculated using the following formulas:

Equation S3.11:

$$\alpha = \frac{\mu^2}{\sigma^2}$$

Equation S3.12:

$$\beta = \frac{\sigma^2}{\mu}$$

where μ represents the deterministic value and σ represents the SE.

Variable 19: Utility for a patient with a coiled aneurysm living with good functional status (mRS=0)

Variable 20: Utility for a patient with a coiled aneurysm living with mild disability (mRS=1-2)

Variable 21: Utility for a patient with a coiled aneurysm living with moderate to severe disability (mRS=3-5)

Variable 19 was derived from the US cross-sectional survey by Fryback et al. [15]. This article found that among 3,844 respondents, their average utility per year as measured by the EQ-5D by age in years was: 45-54: 0.87, 55-64: 0.85, 65-74: 0.86, 75: 0.84. All had a SE of 0.01. The appropriate utility was selected based on the age of patients at each cycle of the model. Variable 20 and 21 were derived from the systematic review by Post et al. [16]. This article found that based on the Time-Trade-Off method, the mean utility for patients with a stroke causing mild disability was 0.72 (Range: [0.71, 0.81]) and with a stroke causing moderate to severe disability was 0.41 (Range: [0.37, 0.71]). Since the ranges were provided in place of the SEs, we estimated the SE by assuming a normal distribution and using the following formula:

Equation S3.13:

$$SE \cong \frac{UB - LB}{4}$$

where UB and LB represent the upper and lower bound of the range respectively.

SEs were therefore calculated to be 0.025 and 0.085 for variables 20 and 21 respectively. Variables 19 to 21 were drawn from truncated normal distributions bounded by 0 and 1. To preserve the relative order of utilities among Variables 19 to 21, the Dirichlet style methodology described for Variables 12 to 14 was followed.

Variable 22: Utility lost from living with the knowledge of having an untreated aneurysm

This decrease in utility was derived from the prospective cohort study by van der Schaaf et al. [17]. The article found using the EQ-5D that patients who developed a recurrent or de novo aneurysm reported a utility which was on average 0.07 (95%CI: [-0.01, 0.15]) lower than matched patients who did not develop a recurrent or de novo aneurysm. The SE was calculated to be 0.04082 using Equation S3.01. Variable 22 was drawn from a beta distribution, a common distribution used for disutilities in economic analyses, as it is inherently bounded between 0 and 1. The values for α and β for this distribution were calculated using Equations S3.02 and S3.03. Patients in the model who had an untreated aneurysm including all *Recanalized* states as well as all *De Novo*$_4$ states would experience this disutility. Additionally, all patients undergoing treatment of a de novo or recanalized aneurysm, including all *Recoiled* states and all remaining *De Novo* states, would also experience this disutility, however, once their aneurysm was treated the disutility would no longer apply.

Variable 23: Utility for a patient that has an aSAH

The utility of patients actively having an aSAH was not found in the published scientific literature. Therefore, we approximated this to be equivalent to the utility associated with moderate to severe disability (Variable 21). In the probabilistic model, Variable 23 and 21 are always equal.

Baseline Mortality Rate:

The baseline rate of mortality was taken from the US National Centre for Health Statistics' CDC WONDER Online Database and compiled from the 57 vital statistics jurisdictions through the Vital Statistics Cooperative Program [3]. This data spanned years 1999 to 2019

and was stratified by age. Both mortality rates as well as SEs by age were extracted. Baseline age-specific mortality rates were drawn from beta distributions, a common distribution type used for probabilities in economic analyses, as it inherently bounds probabilities between 0 and 1. The values for α and β for each beta distribution were calculated using Equations S3.02 and S3.03. For patients in a state that contained an aneurysmal-related cause for mortality, for example dying from a coiling procedure in *De Novo*, the overall mortality rate for that state was always calculated by summing the baseline age-specific mortality rate with the aneurysmal-related mortality rate.

Other Parameters:
The following parameters were not taken from the published scientific literature, rather they were calculated as a function of other parameters in the model. This approach was taken to ensure that the sum of the transitional probabilities associated with each state always equalled 1.

Variable O_1: Probability of maintaining pre-procedural functional status following a coiling procedure

This transitional probability was derived using the following formula:

If pre-procedure status is good (mRS=0):

Equation S3.14a:

$$P_A = 1 - V_1 - V_2 - V_3 - Dead_B$$

If pre-procedure status is mildly disabled (mRS=1-2):

Equation S3.14b:

$$P_A = 1 - V_2 - V_3 - Dead_B$$

If pre-procedure status is moderate to severely disabled (mRS=3-5):

Equation S3.14c:

$$P_A = 1 - V_3 - Dead_B$$

where P_A is the transitional probability of maintaining pre-procedural functional status following a coiling procedure, V_i is Variable i (see Table 1), and $Dead_B$ is the age specific baseline mortality rate.

Variable O_2: Probability of remaining in $Coiled_{status_i}$

This transitional probability was derived using the following formula:

If procedure was within previous 6 years:

Equation S3.15a:

$$\begin{aligned} P_B &= 1 - (V_4 - V_5) - V_5 - V_6 - Dead_B \\ &= 1 - V_4 - V_6 - Dead_B \end{aligned}$$

If procedure was longer than 6 years ago:

Equation S3.15b:

$$P_B = 1 - V_6 - Dead_B$$

where P_B is the transitional probability of remaining in $Coiled_{status_i}$, V_i is Variable i (see Table 1), and $Dead_B$ is the age specific baseline mortality rate.

Variable O_3: Probability of remaining in $Recanalized_{status_i}$

This transitional probability was derived using the following formula:

Equation S3.16:

$$P_C = 1 - V_6 - V_7 - Dead_B$$

where P_C is the transitional probability of remaining in $Recanalized_{status_i}$, V_i is Variable i (see Table 1), and $Dead_B$ is the age specific baseline mortality rate.

Variable O_4: Probability of reaching hospital alive following an aSAH

This transitional probability was derived using the following formula:

Equation S3.17:

$$P_D = 1 - V_8$$

where P_D is the transitional probability of reaching hospital alive following an aSAH and V_i is Variable i (see Table 1).

Variable O_5: Probability of having good functional status (mRS=0) following an aSAH, given patient reached hospital alive

This transitional probability was derived using the following formula:

Equation S3.18:

$$P_E = 1 - V_9 - V_{10} - V_{11} - Dead_B$$

where P_E is the transitional probability of having good functional status (mRS=0) following an aSAH, given patient reached hospital alive, V_i is Variable i (see Table 1), and $Dead_B$ is the age specific baseline mortality rate.

Variable O_6: Probability of remaining in $Post\ SAH_{status}$

This transitional probability was derived using the following formula:

Equation S3.19:

$$P_F = 1 - Dead_B$$

where P_F is the transitional probability of remaining in $Post\ SAH_{status}$ and $Dead_B$ is the age specific baseline mortality rate.

Supplement 4

The following is a list of what is felt to be the main assumptions of the model and their respective justifications.

Assumption S4.01
The model was run for 30 years.
Justification: Instead of running the model for the lifetime of patients, we followed patients for 30 years and stopped the model when all patients were 75. It was felt that after 30 years, all the effects of the HTI would have been realized. As patients age, especially beyond 75, we expect that their other medical conditions would begin to play a larger role in their overall functional status and life expectancy. Moreover, as patients become more advanced in age, they may no longer be candidates or wish to pursue endovascular therapy. Accounting for all these factors would be extremely complex yet would act equally on both treatment arms, thus it would have minimal impact on the ICER or study conclusions. We therefore chose to focus the model on ages 45 to 75, when patients would most likely be offered endovascular treatment and their other medical comorbidities play less of a role in their overall functional status.

Assumption S4.02
In this model, all de novo aneurysms that are identified are treated.
Justification: Because we were unable to estimate on an individual basis which aneurysms would be treated and which would not, and furthermore because we had no objective way to estimate the risk of rupture for aneurysms that were not offered treatment, we chose to have all de novo aneurysms treated. This helped simplify the model and keep the number of health states manageable. We felt justified in making this assumption since it affects each treatment arm equally and thus does not impact the ICER or conclusions of this analysis.

Assumption S4.03
Aneurysms are not clipped in this model, only coiled.
Justification: For the index procedure specifically, this assumption is clearly justified given our population of interest consists only of patients undergoing coil embolization of their UIA, as these are the only patients that can receive the HTI. If a coiled aneurysm recanalizes, both coiling and surgical clipping have been shown to be equally effective treatments [18]. To help simplify the model, we have chosen to use only coiling procedures effectively limiting the number of health states. If clipping were included in the model, these patients would have a lower recanalization rate, however, the fraction of aneurysm that would be clipped would be minimal and would therefore not affect the overall findings of the study. Conversely, a reasonable proportion of de novo aneurysms may receive surgical clipping. Including clipping of de novo aneurysms in the model, however, would affect each treatment arm equally and have no impact on the ICER or study conclusions. Clipping of de novo aneurysms was therefore left out to reduce the number of health states and avoid unnecessary model complexity.

Assumption S4.04
Coiling procedures do not fail in this model.
Justification: 10.3% of coiling procedures do not result in complete aneurysm occlusion [5]. It is unclear, however, if the risk of rupture for these aneurysms is similar to completely occluded

aneurysms that subsequently recanalized. Including partially treated coiled aneurysms would dramatically increase the number of health states and complexity in the model. Because we have no reason to suspect the HTI would affect the rate of partial occlusion when coiling UIA, including this in the model should affect both treatment arms equally and therefore have no impact on the ICER or study conclusions.

Assumption S4.05
Patients are only offered at most 4 endovascular coiling procedures in this model.
Justification: Each additional endovascular procedure added 48 new health states to the model, therefore we limited the number of endovascular procedures to 4 to keep the number of health states manageable. While it is possible that a patient may undergo more than 4 endovascular coiling procedures, it is extremely uncommon. In looking at long-term follow-up data for patients with coiled UIA, Ries et al. found 0 patients that underwent retreatment 4 or more times and only 3 of 342 patients underwent retreatment 3 times [6].

Assumption S4.06
All de novo and recanalized aneurysms in this model are identified and either coiled or managed conservatively; none present as an aSAH. Only known unprotected aneurysms can rupture in this model.
Justification: A small proportion of the recanalized or de novo aneurysms would likely have presented with aSAH prior to diagnosis in a real clinical setting. This proportion would depend on the frequency of patient follow-ups and neurovascular imaging, as well as the waiting time for elective endovascular coiling procedures. Practice patterns vary significantly for both these metrics. Given the average aneurysm rupture risk is 1.4% per year [8] and the amount of time a patient lives with an undiagnosed new or recanalized aneurysm is likely short, including this in the model would add significant complexity without having a large effect on the model results. Furthermore, removing this assumption would result in better outcomes for the HTI arm, as these patients develop fewer recanalizations and would therefore be less at risk of living with an undiagnosed unprotected aneurysm.

Assumption S4.07
Aneurysms in this model can only recanalize within 6 years of coiling and during this time, the recanalization rate is constant. After the 6 years, if they have not recanalized, aneurysms are considered permanently cured.
Justification: In a long-term follow-up study of coiled UIA by Ries et al., recanalizations did not occur more than 77 months following the index coiling procedure, with angiographic follow-up for some patients as long as 132 months [6]. Recanalizations happened closer to the time of the index procedure, with an average of 17.9 months. Given most recanalizations occur prior to 72 months, we felt justified rounding the recanalization window down to 72 months as opposed to 78 (given our cycle length was 6 months). Moreover, because we did not have detailed data on how the recanalization rate changes over time, we assumed a uniform distribution over the 72 months. We know that most recanalizations occur earlier on in the follow-up period, however, given our discount factor is 1.5% the additional delay in costs and QALY this assumption introduces should cause only minimal effects on the ICER.

Assumption S4.08
When an aneurysm is recoiled in this model, the recanalization rate is the same as for the index coiling procedure.
Justification: It is possible that the recanalization rate of aneurysms that are recoiled is different from UIA coiled for the first time. In the study by Ries et al., of the 342 patients with coiled UIA, 33 underwent re-treatment. Of these 33, 4 (12%) underwent retreatment twice, and 3 (9%) underwent retreatment three times [6]. Given how small the sample is here, and how similar the percentages are to the 9.1% retreatment rate we used in our model (Variable 5), we felt it was reasonable to assume recanalization rates are the same, regardless of number of coiling attempts.

Assumption S4.09
Aneurysms in this model that have recanalized are treated if a patient undergoes a coiling procedure for a de novo aneurysm.
Justification: If patients in $Recanalized_i$ develop a de novo aneurysm, they transition to $De\ Novo_i$ and are indistinguishable from patients in $De\ Novo_i$ that transitioned from $Coiled_i$. Once coiled, these patients all transition to $Coiled_{i+1}$. These patients, therefore, no longer have a recanalized aneurysm. We assumed that during the coiling procedure, after the de novo aneurysm was treated, the recanalized aneurysm was also coiled and no longer at risk of rupture. It is possible that some recanalized aneurysms may not have been retreated at this time, however, accounting for this would have drastically increased the number of health states and complexity of the model. We felt justified in this assumption because interventionalists would likely defer recoiling the recanalized aneurysm only if the expected risk of rupture was low.

Assumption S4.10
The HTI in this model does not cause any significant complications.
Justification: In our base case, we explore the use of MSC therapy. MSC therapy has been shown to be very safe in clinical studies, with minimal side-effects and complications, as described in the review by Uccelli et al. on MSC use in neurologic diseases [19]. Complications were uncommon and mainly included fever and infection associated with IV insertion, treated effectively with antibiotics. There were no reported serious adverse events. It was felt that the small cost and brief decrease in QALY associated with these rare complications would not significantly affect the ICER, especially compared to the complications associated with the endovascular procedure itself, and were therefore not included in the model. If an HTI is developed that does impart complications with a reasonable likelihood of significant impact on the costs and QALY, this should be included in the model.

Assumption S4.11
The HTI in this model is only administered at the index procedure and imparts benefit only on the recanalization rate for the first coiling procedure.
Justification: It may be possible for a HTI to be developed that has longstanding benefits, even following a retreatment procedure, in which case this assumption can be removed from the model. We chose to limit the benefits of the HTI to the first coiling procedure only, in order to be more conservative and avoid providing the HTI any undue advantage over standard treatment. We exclusively tested the strategy where the HTI would be administered only at the index procedure. This allowed our results to reflect a direct comparison between standard treatment and HTI use for a single coiling procedure. In future, should there be a need, we can use our model to test an alternative strategy where all coiling procedures utilize the HTI, permitting we have the necessary efficacy data.

Assumption S4.12
The aneurysm recanalization RRR imparted by the HTI in this model acts equally on both aneurysms that would be recoiled as well as aneurysms that would be managed conservatively. Therefore, the proportion of recanalized aneurysms that are treated remains the same with and without the use of the HTI.
Justification: In the absence of clinical data, we assumed the HTI would affect all aneurysms equally. If an HTI is developed and the clinical data shows the HTI prevents recanalization of aneurysms that would have been managed conservatively more than it prevents recanalization of aneurysms that would have been treated (or vice-versa), this will have to be accounted for in the model and new results will be generated.

Assumption S4.13
The aneurysm recanalization RRR imparted by the HTI was drawn from a uniform distribution between 0% and 100% in the model's base case.
Justification: Since no clinical efficacy data is available for the theoretical HTI (including for MSC therapy), this distribution was chosen by the authors. We did not think it would be reasonable to include negative values for the RRR since only HTI with positive RRR would translate to clinical practice. While this assumption is not founded on clinical evidence, it is only used in the base case, and the main results of the study use all values of RRR, thus removing this assumption from the model.

Assumption S4.14
In the EVPPI calculation, the aneurysm recanalization RRR imparted by the HTI was drawn from a normal distribution bounded by 0 and 1 with a mean of 50% and SD of 15%.
Justification: Since no clinical efficacy data is available for the theoretical HTI (including for MSC therapy), this distribution was chosen by the authors. We were unable to perform this calculation without specifying at least some expected value and distribution for the RRR. The values presented in the paper for EVPPI are therefore conditional on the RRR distribution, and the calculation must be repeated if a HTI RRR expected value and distribution are not similar to the one used here.

Assumption S4.15
Following an aSAH, patients in this model can no longer develop recanalized or de novo aneurysms.
Justification: To allow for patients with aSAH to develop recanalized or de novo aneurysms, while still allowing a maximum of four endovascular coiling procedures would have dramatically increased the number of health states and complexity of the model. While we acknowledge that some aSAH survivors do undergo repeat procedures, since the total number of patients in our model that develop aSAH is very low (only 1.4% of unprotected aneurysms develop aSAH per year and ~40% die either immediately or in hospital), making this assumption allowed the model to be much simpler while having minimal effect on the ICER and overall study results.

Supplement 5

The following is a detailed description of how the simulations in this study were performed.

To determine the total costs and total QALY in a Markov model, all costs and QALY for each state are calculated and summed across all the states in a given cycle. The costs and QALY for each cycle are then discounted appropriately and added together to obtain the total costs and QALY for the model. Totals are then compared between the standard treatment arm and the HTI arm to determine whether the use of the HTI is cost-effective.

Iteration Simulation
In the first iteration, all variables were drawn from their respective distributions and used in the Markov model to calculate the total discounted costs and QALY for both the arm that received the HTI as well as the arm that received standard treatment. These values were recorded. Another iteration was completed where new values of each variable were again drawn from their respective distributions and used in the Markov model to generate total discounted costs and QALY. This process was completed x number of times. Once all x iterations were complete, all iterations were averaged together to generate the average total discounted costs and average total discounted QALY for both treatment arms. These averages were used to calculate the ICER using the following formula:

Equation S5.01:

$$ICER = \frac{Cost_{HTI} - Cost_{Stnd}}{QALY_{HTI} - QALY_{Stnd}}$$

where $ICER$ is the incremental cost-effectiveness ratio, $Cost_{HTI}$ is the average total discounted costs for the arm that received the HTI, $Cost_{Stnd}$ is the average total discounted costs for the arm that received standard care, $QALY_{HTI}$ is the average total discounted QALY for the arm that received the HTI, and $QALY_{Stnd}$ is the average total discounted QALY for the arm that received standard care.

We varied x from 10 to 500 in increments of 10, from 500 to 1,000 in increments of 100, from 1,000 to 10,000 in increments of 1,000, and finally 20,000. All values of x were run 10 times each, except for $x = 10{,}000$ and $x = 20{,}000$, which were repeated 20 times each. The SD of the ICER for each value of x was then calculated. Looking at the SD plotted over the values of x, the SD appears to stabilize at ~ 7,000 iteration (Fig S5.01). Therefore, for all subsequent simulations, 7,000 iterations were used for each run of the model.

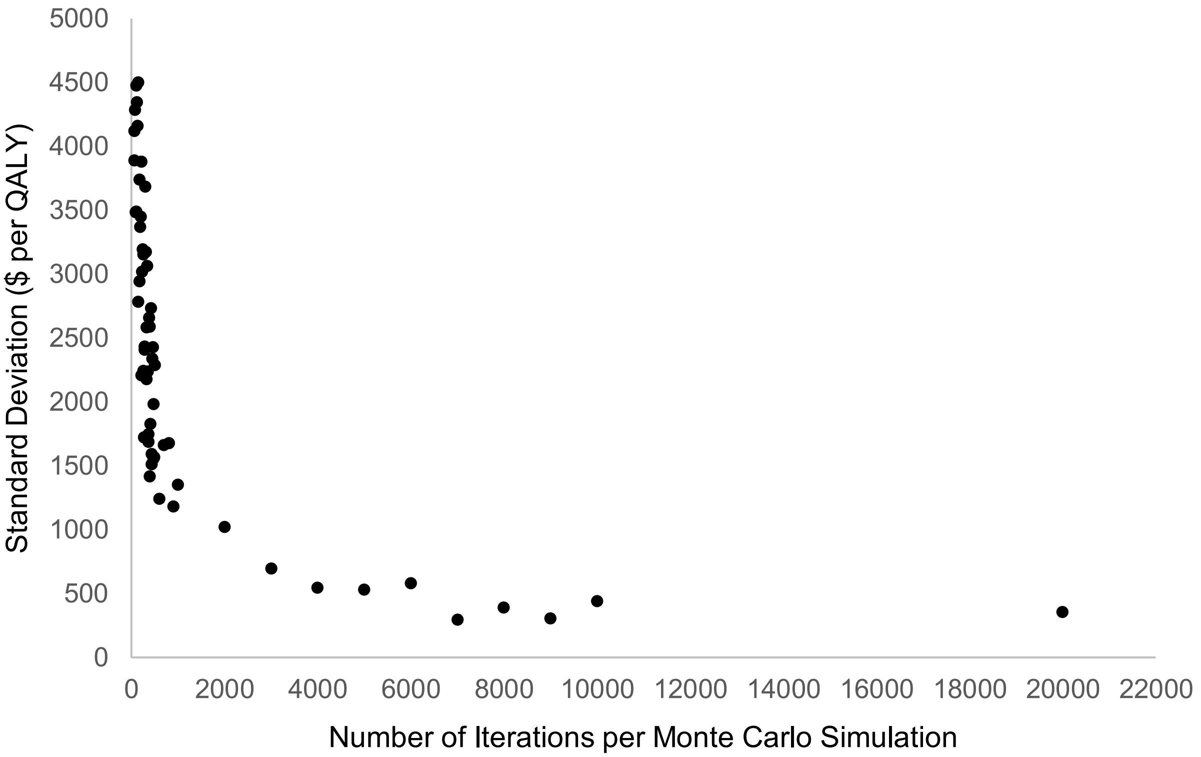

Fig S5.01. Determination of optimal number of iterations for probabilistic Monte Carlo simulation

Note: All currency represented in 2018 United States dollars.
Abbreviations: ICER – incremental cost-effectiveness ratio; QALY – quality-adjusted life-years.

Base Case

To perform the base case probabilistic analysis, a Monte Carlo simulation using 7,000 iterations was completed. The average total discounted costs and average total discounted QALY for both treatment arms, as well as the ICER calculated using Equation S5.01 were all recorded. The probability that using the HTI would be cost-effective as well as the EVPI was calculated and recorded at multiple threshold levels varying from $0 per QALY to $150,000 per QALY (see CEAC and EVPI below). This simulation was repeated 100 times. Using the results from all 100 runs, the average values, SE, and 95% credible intervals (CrI) for the ICER, the probability of cost-effectiveness (over multiple thresholds), and the EVPI (over multiple thresholds) were calculated and recorded. SE and 95% CrI for the total discounted costs and the total discounted QALY of each treatment arm were calculated using the 7,000 iterations from the final (100th) run.

CEAC

Once the Monte Carlo simulation with 7,000 iterations was completed (see Base Case above), the NMB in each iteration for the HTI arm and the standard treatment arm were calculated using the following formula:

Equation S5.02:

$$NMB_i = QALY_i \times T - Cost_i$$

where T is the threshold value (in 2018 USD per QALY) and i is the treatment arm (treatment with the HTI or standard treatment).

Once the NMB for each treatment arm was calculated, the probability that HTI use was cost-effective was calculated using the following formula:

Equation S5.03:

$$P_{HTI} = \frac{N_{HTI}}{N_{Total}}$$

where P_{HTI} is the probability that HTI use is cost-effective, N_{HTI} is the number of iterations the NMB when using the HTI was greater than the NMB for standard treatment, and N_{Total} is the total number of iterations (7,000 in this case).

This process of calculating NMB_i and P_{HTI} using Equations S5.02 and S5.03 was repeated several times, with threshold levels varying from 0 to 1,000 in increments of 100, from 1,000 to 30,000 in increments of 1,000, and from 30,000 to 150,000 in increments of 5,000, where all threshold values are in 2018 USD per QALY. Fig S5.02 illustrates the CEAC at all threshold levels tested.

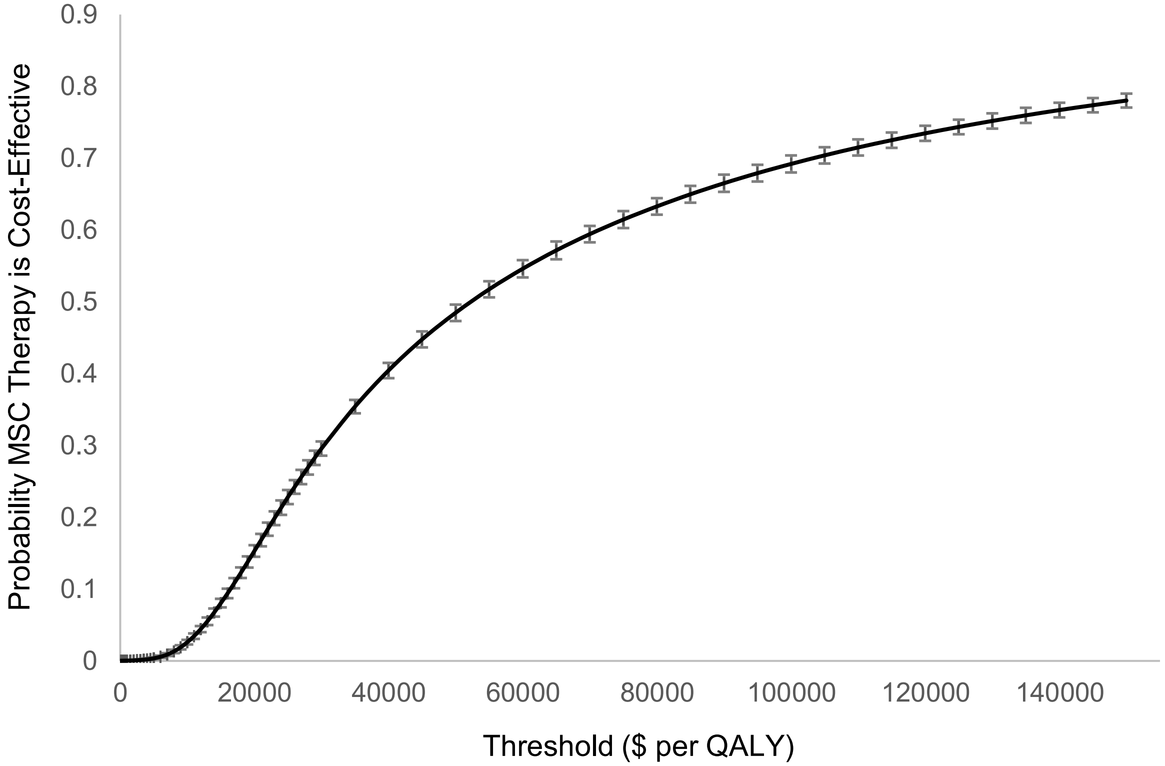

Fig S5.02. Cost-effectiveness acceptability curve

Note: All currency represented in 2018 United States dollars. Error bars represent the 95% credible interval.
Abbreviations: MSC – mesenchymal stem cell; QALY – quality-adjusted life-years.

EVPI

Once the Monte Carlo simulation with 7,000 iterations was completed (see Base Case above) and the NMB_i was calculated for each iteration (see CEAC above), the treatment arm with the highest probability of cost-effectiveness was identified. This was carried out using a specific threshold level. If $P_{HTI} > 0.5$ the HTI arm was chosen to more likely be cost-effective and if

$P_{HTI} \leq 0.5$ the standard treatment arm was chosen. The arm which was chosen to more likely be cost-effective was termed the "expected best option". For each iteration k, the NMB_i with the higher value (the NMB for either the HTI arm or the standard treatment arm) was also identified and termed the "true best option". The EVPI for each iteration was then calculated using the following formula:

Equation S5.04:

$$EVPI_k = NMB_T - NMB_E$$

where $EVPI_k$ is the expected net benefit gained by resolving the uncertainty associated with all parameters used in the model for iteration k, NMB_T is the NMB of the true best option for iteration k, and NMB_E is the NMB in iteration k for the expected best option. Note, the expected best option will be the same for all iterations (for a given threshold), while the true best option may change between iterations.

EVPI per procedure was then calculated by averaging the $EVPI_k$ across all 7,000 iterations. The SD and 95%CrI were also calculated. Assuming the HTI would be used in 15,925 procedures per year [20] for a total of 10 years, with a discount rate of 1.5%, the total EVPI was calculated using the following formula:

Equation S5.05:

$$EVPI_T = EVPI_P \cdot N_P \cdot \frac{1-(1+r)^{-i}}{r}$$

where $EVPI_T$ is the total EVPI, $EVPI_P$ is the per procedure EVPI, N_P is the number of procedures performed per year (15,925), r is the discount rate (1.5%), and i is the time in years the HTI is assumed to be used (10 years).

We then calculated the 95%CrI for $EVPI_T$ using the per procedure EVPI 95%CrI. This process was repeated for all values of threshold described in the CEAC simulation (see CEAC above). Fig S5.03 illustrates how the EVPI varies with changes in the threshold.

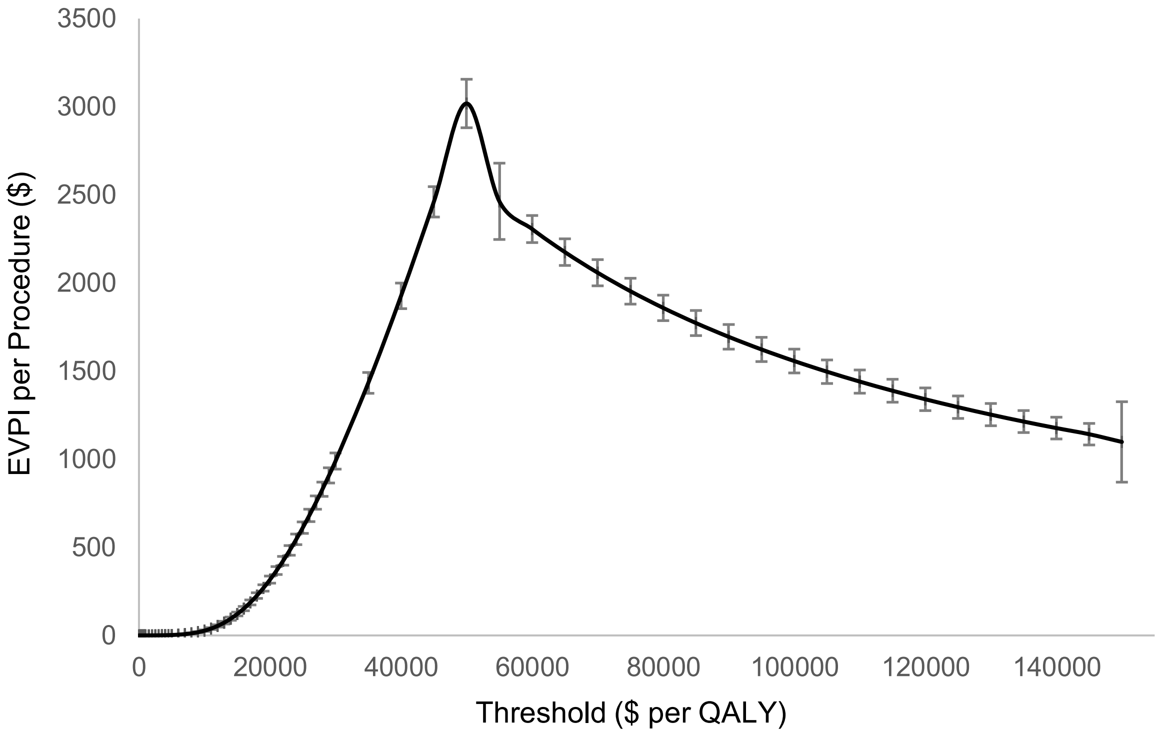

Fig S5.03. EVPI over changes in threshold

Note: All currency represented in 2018 United States dollars. Error bars represent the 95% credible interval.
Abbreviations: EVPI – expected benefit of perfect information; QALY – quality-adjusted life-years.

Sensitivity Analysis

To perform the one-way sensitivity analysis, we set each model parameter in turn to two standard errors below the mean, followed by two standard errors above the mean. Exceptions to this approach included the HTI efficacy (Variable 24) and cost (Variable 25), which were not tested in the sensitivity analysis, and the discount rate for cost and QALY which was tested at 0%, 3%, and 5% as per national guidelines [2, 21]. For some parameters, however, two standard errors were not used for the following reasons:

Healthcare cost of a coiling procedure without complications (Variable 12):
Lower Value: This cost was not allowed to be negative and was therefore bounded by 0.
Upper Value: This cost was not allowed to be greater than the healthcare cost of a coiling procedure with complications leading to functional disability (Variable 13) and was therefore bounded by the probabilistic value of Variable 13.

Healthcare cost of a coiling procedure with complications leading to functional disability (Variable 13):
Lower Value: This cost was not allowed to be lower than the healthcare cost of a coiling procedure without complications (Variable 12) and therefore bounded by the probabilistic value of Variable 12.
Upper Value: This cost was not allowed to be greater than the healthcare cost of a coiling procedure with complications leading to patient death (Variable 14) and was therefore bounded by the probabilistic value of Variable 14.

Healthcare cost of a coiling procedure with complications leading to patient death (Variable 14):
Lower Value: This cost was not allowed to be lower than the healthcare cost of a coiling procedure with complications leading to functional disability (Variable 13) and therefore bounded by the probabilistic value of Variable 13.

Healthcare cost for a patient with a coiled aneurysm living with good functional status (mRS=0) (Variable 15):
Upper Value: This cost was not allowed to be greater than the healthcare cost for a patient with a coiled aneurysm living with mild disability (mRS=1-2) (Variable 16) and was therefore bounded by the probabilistic value of Variable 16.

Healthcare cost for a patient with a coiled aneurysm living with mild disability (mRS=1-2) (Variable 16):
Lower Value: This cost was not allowed to be lower than the healthcare cost for a patient with a coiled aneurysm living with good functional status (mRS=0) (Variable 15) and therefore bounded by the probabilistic value of Variable 15.
Upper Value: This cost was not allowed to be greater than the healthcare cost for a patient with a coiled aneurysm living with moderate to severe disability (mRS=3-5) (Variable 17) and was therefore bounded by the probabilistic value of Variable 17.

Similar bounds which maintained parameter face validity and the integrity of the Dirichlet-style distributions were also imposed on all other relevant parameter values, however, only the bounds mentioned above needed to be invoked.

For each value tested, a Monte Carlo simulation with 7,000 iterations was completed and average total discounted costs and average total discounted QALY for both treatment arms were recorded. The ICER for each Monte Carlo simulation was then calculated using Equation S5.01. For each model parameter, the absolute difference between the ICER in the base case and the ICER for the lower and upper values were calculated and added together to find the "total change in ICER". Model parameters were then arranged in order from largest to least total change in ICER. All parameters with total change in ICER less than or equal to the range of the base case ICER 95%CrI ($1,801.81 per QALY) were removed. The remaining parameter results were plotted in a Tornado diagram (Fig 3).

HTI Cost-Elasticity
To perform the HTI cost-elasticity probabilistic analysis, we began with a Monte Carlo simulation with 700,000 iterations. In this simulation, as in the base case, the aneurysm recanalization RRR imparted by the HTI (Variable 24) was drawn from a uniform distribution bounded by 0% and 100%. However, we set the HTI cost (Variable 25) to $0, such that all total costs derived from the model would not include the HTI cost. For each iteration, we recorded total discounted costs and total discounted QALY for both treatment arms, as well as the RRR drawn. Each RRR was then rounded to the nearest 1%.

NMB_i for each treatment arm for all iterations was then calculated using Equation S5.02 at a specific threshold level. A rounded RRR value was then selected, and for only the iterations

which used this RRR, P_{HTI} was calculated using Equation S5.03. Because we set the HTI cost to \$0 and because all HTI costs were incurred at a single point in time at the beginning of the model, we were able to see how P_{HTI} (for a specific RRR value) changed with changes in the HTI cost by simply subtracting HTI cost from NMB_{HTI}. The HTI cost which resulted in a $P_{HTI} = 0.5$ was identified computationally using the “Goal Seek” function in Microsoft Excel® (Version 16.20) and recorded. This HTI costs was termed the “maximum willingness-to-pay” for the HTI at the given RRR (and threshold). The process was repeated for all values of RRR. Once the maximum willingness to pay for all levels of RRR was identified, the process was repeated using a different threshold level. Thresholds of \$50,000, \$100,000, and \$150,000 per QALY were tested.

Scenario Analyses
To perform the scenario analyses, the methodology used in the base case was followed (see Base Case above). However, instead of drawing the HTI efficacy (Variable 24) from a uniform distribution between 0% and 100%, normal distributions bounded by 0% and 100%, with means (SD) of 10% (2%), 30% (5%), and 50% (15%) were used.

EVPPI
To estimate the expected benefit associated with resolving the uncertainty around the baseline rate of aneurysm recanalization, we first performed a Monte Carlo simulation with 7,000 iterations. For this simulation, the HTI efficacy (Variable 24) was drawn from a normal distribution bounded by 0% and 100%, with a mean (SD) of 50% (15%). In terms of the baseline rate of aneurysm recanalization (Variable 4), we drew a single value from Variable 4’s base case distribution and used this same value for Variable 4 in all 7,000 iterations. Once the Monte Carlo simulation was completed, we recorded the value of Variable 4 used, as well as the average total discounted costs and average total discounted QALY for each treatment arm. We then calculated the NMB_i for each treatment arm using Equations S5.01 and S5.02 at threshold levels of \$50,000, \$100,000, and \$150,000 per QALY. The average NMB_i for both treatment arms across all 7,000 iterations was calculated and recorded. P_{HTI} was also calculated for each threshold level using Equation S5.03 and recorded.

This entire process was repeated 1,000 times. The P_{HTI} was then averaged across all 1,000 runs at each threshold level. Similar to the process followed in the EVPI calculation (see EVPI above), if the average $P_{HTI} > 0.5$, the HTI arm was chosen to more likely be cost-effective (for a given threshold) and if $P_{HTI} \leq 0.5$ the standard treatment arm was chosen. The arm which was chosen to more likely be cost-effective was termed the “expected best option”. For each of the 1,000 runs, the treatment arm with the higher NMB was identified and termed the “true best option”. The EVPPI for each run was then calculated using the following formula:

Equation S5.06:

$$EVPPI_j = NMB_T - NMB_E$$

where $EVPPI_j$ is the expected net benefit gained by resolving the uncertainty associated with the baseline aneurysm recanalization rate for run j, NMB_T is the NMB of the true best option for run j, and NMB_E is the NMB of the expected best option for run j. Note, the expected best option will be the same for all runs (for a given threshold), while the true best option may change between runs.

EVPPI per procedure was then calculated by averaging $EVPPI_j$ across all 1,000 runs. The SD and 95%CrI were also calculated. Assuming the HTI would be used in 15,925 procedures per year [20] for a total of 10 years, with a discount rate of 1.5%, the total EVPPI was calculated using the following formula:

Equation S5.07:

$$EVPPI_T = EVPPI_P \cdot N_P \cdot \frac{1-(1+r)^{-i}}{r}$$

where $EVPPI_T$ is the total EVPPI, $EVPPI_P$ is the per procedure EVPPI, N_P is the number of procedures performed per year (15,925), r is the discount rate (1.5%), and i is the time in years the HTI is assumed to be used (10 years).

We then calculated the 95%CrI for $EVPPI_T$ using the per procedure EVPPI 95%CrI. This process was repeated for each threshold level.

References

1. Husereau D, Drummond M, Petrou S, Carswell C, Moher D, Greenberg D, et al. Consolidated Health Economic Evaluation Reporting Standards (CHEERS) statement. Bmj Br Medical J. 2013;346(mar25 1):f1049.
2. Sanders GD, Neumann PJ, Basu A, Brock DW, Feeny D, Krahn M, et al. Recommendations for Conduct, Methodological Practices, and Reporting of Cost-effectiveness Analyses: Second Panel on Cost-Effectiveness in Health and Medicine. JAMA. 2016;316(10):1093-103.
3. Centers for Disease Control and Prevention. Underyling Cause of Death, 1999-2019, Available from: http://wonder.cdc.gov/ucd-icd10.html.
4. Lanterna LA, Tredici G, Dimitrov BD, Biroli F. Treatment of Unruptured Cerebral Aneurysms by Embolization with Guglielmi Detachable Coils: Case-fatality, Morbidity, and Effectiveness in Preventing Bleeding—A Systematic Review of the Literature. Neurosurgery. 2004;55(4):767 - 78.
5. Naggara ON, White PM, Guilbert F, Roy D, Weill A, Raymond J. Endovascular treatment of intracranial unruptured aneurysms: systematic review and meta-analysis of the literature on safety and efficacy. Radiology. 2010;256(3):887 - 97.
6. Ries T, Siemonsen S, Thomalla G, Grzyska U, Zeumer H, Fiehler J. Long-Term Follow-Up of Cerebral Aneurysms after Endovascular Therapy Prediction and Outcome of Retreatment. American Journal of Neuroradiology. 2007;28(9):1755 - 61.
7. Giordan E, Lanzino G, Rangel-Castilla L, Murad MH, Brinjikji W. Risk of de novo aneurysm formation in patients with a prior diagnosis of ruptured or unruptured aneurysm: systematic review and meta-analysis. Journal of Neurosurgery. 2018;25:1 - 11.
8. Greving JP, Wermer MJH, Brown RD, Morita A, Juvela S, Yonekura M, et al. Development of the PHASES score for prediction of risk of rupture of intracranial aneurysms: a pooled analysis of six prospective cohort studies. Lancet Neurology. 2014;13(1):59 - 66.
9. Huang J, Gelder JMv. The probability of sudden death from rupture of intracranial aneurysms: a meta-analysis. Neurosurgery. 2002;51(5):1101 - 5- discussion 5-7.
10. Qureshi AI, Suri MFK, Nasar A, Kirmani JF, Ezzeddine MA, Divani AA, et al. Changes in Cost and Outcome Among US Patients With Stroke Hospitalized in 1990 to 1991 and Those Hospitalized in 2000 to 2001. Stroke. 2007;38(7):2180 - 4.
11. Hop JW, Rinkel GJ, Algra A, Gijn Jv. Case-fatality rates and functional outcome after subarachnoid hemorrhage: a systematic review. Stroke. 1997;28(3):660 - 4.
12. Brinjikji W, Kallmes DF, Lanzino G, Cloft HJ. Hospitalization Costs for Endovascular and Surgical Treatment of Unruptured Cerebral Aneurysms in the United States Are Substantially Higher Than Medicare Payments. American Journal of Neuroradiology. 2012;33(1):49 - 51.
13. Shemilt I, Thomas J, Morciano M. A web-based tool for adjusting costs to a specific target currency and price year. Évid Policy J Res Debate Pract. 2010;6(1):51-9.
14. Shireman TI, Wang K, Saver JL, Goyal M, Bonafe A, Diener H-C, et al. Cost-Effectiveness of Solitaire Stent Retriever Thrombectomy for Acute Ischemic Stroke: Results From the SWIFT-PRIME Trial (Solitaire With the Intention for Thrombectomy as Primary Endovascular Treatment for Acute Ischemic Stroke). Stroke. 2017;48(2):379 - 87.

15. Fryback DG, Dunham NC, Palta M, Hanmer J, Buechner J, Cherepanov D, et al. US Norms for Six Generic Health-Related Quality-of-Life Indexes From the National Health Measurement Study. Medical Care. 2007;45(12):1162 - 70.
16. Post PN, Stiggelbout AM, Wakker PP. The utility of health states after stroke: a systematic review of the literature. Stroke. 2001;32(6):1425 - 9.
17. Schaaf ICvd, Wermer MJH, Velthuis BK, Buskens E, Bossuyt PMM, Rinkel GJE. Psychosocial impact of finding small aneurysms that are left untreated in patients previously operated on for ruptured aneurysms. Journal of Neurology, Neurosurgery & Psychiatry. 2006;77(6):748 - 52.
18. Petr O, Brinjikji W, Thomé C, Lanzino G. Safety and efficacy of microsurgical treatment of previously coiled aneurysms: a systematic review and meta-analysis. Acta Neurochirurgica. 2015;157(10):1623 - 32.
19. Uccelli A, Laroni A, Freedman MS. Mesenchymal stem cells for the treatment of multiple sclerosis and other neurological diseases. Lancet Neurology. 2011;10(7):649 - 56.
20. Huang MC, Baaj AA, Downes K, Youssef AS, Sauvageau E, Loveren HRv, et al. Paradoxical Trends in the Management of Unruptured Cerebral Aneurysms in the United States. Stroke. 2011;42(6):1730 - 5.
21. Canadian Agency for Drugs Technologies in Health. Guidelines for economic evaluation of health technologies, Canada, 4th Ed. 2017.

Reference 3 – remove square bracket
